# Data visualisation approaches for component network meta-analysis: visualising the data structure

**DOI:** 10.1186/s12874-023-02026-z

**Published:** 2023-09-15

**Authors:** Suzanne C. Freeman, Elnaz Saeedi, José M. Ordóñez-Mena, Clareece R. Nevill, Jamie Hartmann-Boyce, Deborah M. Caldwell, Nicky J. Welton, Nicola J. Cooper, Alex J. Sutton

**Affiliations:** 1 Biostatistics Research Group, Department of Population Health Sciences, University of Leicester, Leicester, UK; 2 NIHR Complex Reviews Support Unit, University of Leicester and University of Glasgow, Leicester, UK; 3 Nuffield Department of Primary Care Health Sciences, University of Oxford, Oxford, UK; 4 Population Health Sciences, Bristol Medical School, University of Bristol, Bristol, UK

**Keywords:** Component network meta-analysis, Data visualisation, Meta-analysis, Presentational tools, Graphical displays, Multicomponent interventions, Complex interventions

## Abstract

**Background:**

Health and social care interventions are often complex and can be decomposed into multiple components. Multicomponent interventions are often evaluated in randomised controlled trials. Across trials, interventions often have components in common which are given alongside other components which differ across trials. Multicomponent interventions can be synthesised using component NMA (CNMA). CNMA is limited by the structure of the available evidence, but it is not always straightforward to visualise such complex evidence networks. The aim of this paper is to develop tools to visualise the structure of complex evidence networks to support CNMA.

**Methods:**

We performed a citation review of two key CNMA methods papers to identify existing published CNMA analyses and reviewed how they graphically represent intervention complexity and comparisons across trials. Building on identified shortcomings of existing visualisation approaches, we propose three approaches to standardise visualising the data structure and/or availability of data: CNMA-UpSet plot, CNMA heat map, CNMA-circle plot. We use a motivating example to illustrate these plots.

**Results:**

We identified 34 articles reporting CNMAs. A network diagram was the most common plot type used to visualise the data structure for CNMA (26/34 papers), but was unable to express the complex data structures and large number of components and potential combinations of components associated with CNMA. Therefore, we focused visualisation development around representing the data structure of a CNMA more completely. The CNMA-UpSet plot presents arm-level data and is suitable for networks with large numbers of components or combinations of components. Heat maps can be utilised to inform decisions about which pairwise interactions to consider for inclusion in a CNMA model. The CNMA-circle plot visualises the combinations of components which differ between trial arms and offers flexibility in presenting additional information such as the number of patients experiencing the outcome of interest in each arm.

**Conclusions:**

As CNMA becomes more widely used for the evaluation of multicomponent interventions, the novel CNMA-specific visualisations presented in this paper, which improve on the limitations of existing visualisations, will be important to aid understanding of the complex data structure and facilitate interpretation of the CNMA results.

**Supplementary Information:**

The online version contains supplementary material available at 10.1186/s12874-023-02026-z.

## Background

**Fig. 1:**
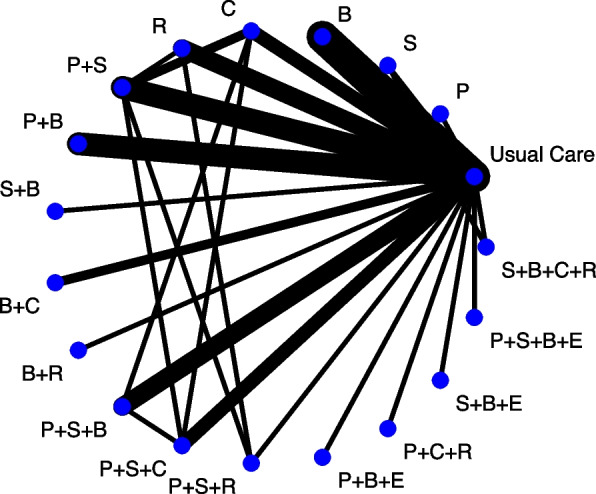
Network diagram at intervention level for the psychological preparations dataset [3]. E = emotion-focused techniques, R = relaxation, C = cognitive interventions, S = sensory information, P = procedural information, B = behavioural instruction

Health and social care interventions are often complex and can be decomposed into multiple components; for example, smoking cessation and weight management interventions may consist of different forms of motivational and behavioural components and may be delivered in different formats, by different providers, at different settings, and/or different intensities [1, 2]. These multicomponent interventions are often evaluated in randomised controlled trials (RCTs). Across trials, interventions often have components in common which are given alongside other components which differ across trials. For example, one trial of psychological interventions for adults undergoing surgery [3], with respective network diagram recreated in Fig. 1, may compare behavioural instruction (B) together with relaxation (R) against usual care whilst another compares behavioural instruction (B) together with cognitive intervention (C) against usual care. Multicomponent interventions are often considered ‘complex’ due to the number of interacting components [4–6]. The Cochrane Handbook goes one step further considering intervention complexity as a spectrum with all interventions having some aspect of complexity [7].

Pairwise meta-analysis (MA) allows for comparisons between two healthcare interventions. For multicomponent interventions this can result in ‘lumping’ different interventions together for comparison to ‘usual care’. Pairwise MA can answer the question “is any form of intervention more effective than usual care?” but is unable to identify and distinguish between components which may be driving the effectiveness or harming the effectiveness of an intervention.

At the other end of the scale to the ‘lumping’ approach of pairwise MA is the ‘splitting’ approach of network meta-analysis (NMA). NMA treats each unique combination of components as a separate node in the network (as in Fig. 1). However, this can result in a network with many nodes but few trials connecting the nodes to anything other than ‘usual care’ (and networks may sometimes be disconnected altogether). This can lead to fitting a model with many parameters but few trials contributing to their estimation, which results in large amounts of uncertainty surrounding the intervention effects and provides little insight into the relative contribution of individual components or how they interact with each other.

An alternative approach for synthesising trials of multicomponent interventions is component NMA (CNMA) where the model estimates the effect of each component. In a CNMA, the estimation of each component can be informed by multiple study designs. For example, for the effect of the single component relaxation in Fig. 1, the following study designs are contributing to the estimation:

1. Studies in which the component alone is compared against no component (e.g., Relaxation (R) versus usual care)
2. Studies in which the component is administered in combination with one or more components (e.g., Procedural information + Sensory information + Relaxation (P + S + R) vs Procedural information + Sensory information (P + S))
3. Studies in which the component is administered in combination with one or more components against no components (e.g., Behavioural instruction + Relaxation (B + R) vs usual care)

Furthermore, using the properties of NMA, we can use indirect evidence to also contribute to the estimation of the effect of relaxation (R). For example, even though we have no trials comparing behavioural instruction + relaxation (B + R) vs behavioural instruction (B), we can use comparisons of B + R vs usual care and B vs usual care, to indirectly estimate the effect of R, and use that indirect effect to contribute to the estimation of the effect of R. Thus, compared to NMA, CNMA can reduce uncertainty around estimates of effectiveness.

CNMA can predict the effectiveness for any combination of components including combinations not previously included in trials. In the psychological interventions example presented in this paper, CNMA can predict the effectiveness of combination behavioural instruction, relaxation and emotion-focused techniques (B + R + E), a combination of components that cannot be estimated from the NMA displayed in Fig. 1. Thus, in addition to desirable analytic properties, CNMA has the potential to answer the most relevant questions for clinical practice such as “which components are the most effective?”, “how can interventions be optimised to include the most effective components only?”, or “can an ineffective component be removed to reduce the cost of the intervention?”. The answers to these kinds of questions can inform the design of future RCTs and the implementation of more cost-effective interventions, across all disease areas.

CNMA was first proposed in 2009 by Welton et al. [8] as a series of network multivariable meta-regression models. The simplest model is the additive effects model in which the effect of a combination of components is assumed equal to the sum of the effects of the individual components. Synergistic or antagonistic effects of components given in combination are accounted for by extending the additive effects model to allow for interactions between pairs of components. This model can be extended further to include 3-way and then 4-way interactions, etc. (the full interaction model is a standard NMA model in which each unique combination of components is treated as a separate node in the network). In recent years there has been an increase in the use of this CNMA modelling approach, for evaluating both public health interventions and for combinations of drug treatment, along with further methodological development, e.g. [1, 3, 9–13]. However, it remains that in some cases, not all components can be estimated uniquely [14]. For example, if components A and B are always given together then the additive effects model cannot distinguish the effect of component A separate to the effect of component B. Furthermore, fitting models including interactions between components requires rich evidence structures so the choice of models that can be fitted may be limited. Therefore, it is important to be able to visualise the evidence available to guide model choice.

This paper considers approaches to the visualisation of CNMA data structures to aid understanding of the available evidence/data and communication of the results of this useful but relatively complex modelling approach to evidence synthesis. This was motivated through the authors conducting several CNMA analyses and struggling to adequately present the structure of the data, e.g. smoking cessation review [1]. A recent paper reviewing meta-analysis visualisations identified 208 graphical displays for meta-analysis and systematic reviews [15]. The graphical displays were categorised into a taxonomy of 11 main classes evaluating 24 graph functionality features. One-hundred-and-fourteen distinct plots were identified with 94 variants. The most prevalent class was NMA (45 displays). It is perhaps not a surprise that NMA has spawned a number of specific plots considering the added complexity compared to pairwise meta-analysis. Whilst CNMA is intrinsically linked to NMA there are significant differences between the approaches; however, no CNMA specific visualisations were identified. For example, whilst a standard NMA network plot (like Fig. 1) can present the intervention combinations administered in the relevant trials, this information quickly becomes difficult to digest as the number of component combinations trialled increases. Therefore, distinct graphical approaches for CNMA are needed.

In this paper, we consider each unique combination of components to be an intervention and interventions may consist of a single component or multiple components given in combination. To aid clarity, we avoid using the term treatment as this is often used interchangeably, and without distinction, to refer to either components or interventions.

The aims of this paper are to understand what visualisations authors are currently using to report CNMA, to identify where ‘gaps’ exist with current visualisations and to propose existing and novel plots for visualising CNMA to address those gaps. We focus on plots that are useful for understanding the data structure and exploratory analyses, briefly consider plots for presenting results, and consider the applicability of plots to different sized networks (something we term scalability).

We start, in Review of current approaches used for visualising CNMA Section, by identifying which visualisations are currently used to display the availability of data and/or the data structure and the results of CNMA and draw conclusions to inform the development of the novel plots—CNMA-UpSet plot, CNMA heat map and CNMA-circle plot – described in Novel plots Section. We finish with a discussion in Discussion Section.

## Review of current approaches used for visualising CNMA

### Literature identification methods

To understand which visualisations are currently used for reporting CNMA, we performed a citation review of two key papers. The first key paper we considered is the paper by Welton et al. [8], which first proposed CNMA in 2009. In this paper, CNMA models are fitted under the Bayesian framework and code is provided for fitting the models in WinBUGS. The second key paper we considered is the paper by Rücker et al*.* [9], which is the first paper to propose the use of CNMA under the frequentist framework and contains details on fitting CNMA models in R using the netmeta package [16].

The citation review was conducted in Google Scholar and Web of Science on the 26^th^ January 2022. Papers were eligible for inclusion in our review if (in addition to citing one or both of the key papers) they applied CNMA models to a dataset, reported the results and were written in English. In addition, we included the Welton et al*.* [8] and Rücker et al*.* [9] papers themselves in our review.

We extracted information on title, first author, year of publication and network size (number of components, number of interventions (i.e. the number of unique combinations of components trialled), number of trials) as well as information on the types of figures and the information presented in figures and tables. We searched and extracted data from both the main manuscript and any online appendices.

To identify ‘gaps’ that exist with current visualisations we considered whether results presented in tables could be presented using graphical approaches identified through our citation review or whether the tables contained additional results not previously displayed in a visual manner.

### Review of visualisations identified (with discussion)

The citation review identified 277 unique articles citing either the Welton [8] or the Rücker paper [9]. We excluded 207 articles that neither applied the CNMA models to a dataset nor reported the results, 22 articles not written in English and 14 articles we were unable to access. Thirty-four papers (including Welton et al*.* [8] and Rücker et al*.* [9]) were identified as meeting the inclusion criteria for this review (Table 1). Ten of these thirty-four papers (29%) included at least one author of this paper.

**Table 1:** Study characteristics

First Author	Publication Year	Network Plot	Summary Forest Plot	Ranking Components Plot	Other Plot (which)	Component Effects Table	Intervention Effects Table	Ranking Components Table	Other Table (which)
Rücker [9]	2019	No	Intervention level	No	No	Yes	No	No	No
Rücker [12]	2020	Yes	Intervention level	No	Yes (Line plot for treatment effects from multiple models)	No	No	Yes	Yes (Model fit)
Mills [13]	2011	Yes	Intervention level	No	No	Yes	Yes	Yes	No
Pompoli [17]	2018	Yes	No	No	Yes (Risk of bias)	Yes	No	No	No
Miklowitz [18]	2020	Yes	No	No	No	Yes	Yes	No	Yes (Risk of bias)
Mills [19]	2012	Yes	No	No	No	Yes	Yes	No	No
Shi [20]	2020	Yes	Intervention level	Yes	Yes (Risk of bias)	No	No	No	Yes (Risk of bias, meta-regression)
Caldwell [21]	2016	Yes	Component level	No	No	No	No	No	No
Freeman [3]	2018	Yes	Component & Intervention level	Yes	Yes (Line plot showing how outcome changes over covariate values)	Yes	No	No	Yes (Model fit)
Kabboul [22]	2018	No	No	No	Yes (Risk of bias, histogram of posterior distributions)	Yes	No	No	Yes (Model fit)
Hartmann-Boyce [1]	2021	No	Component level	No	Yes (Contour plot for model fit, risk of bias)	Yes	No	No	No
López-López [23]	2019	Yes	Component & Intervention level	No	Yes (Risk of bias)	No	No	No	Yes (Model fit)
Riemsma [24]	2011	Yes	No	No	No	Yes	Yes	No	No
Melton [25]	2020	Yes	No	No	Yes (Risk of bias)	Yes	No	No	Yes (Model fit)
Madan [11]	2014	Yes	No	No	No	Yes	No	No	Yes (Model fit)
Chen [26]	2012	Yes	No	No	Yes (Funnel plot, caterpillar plot)	No	Yes	No	No
Weibel [27]	2020	Yes	Intervention level	No	Yes (Risk of bias)	No	No	No	No
Danko [28]	2018	No	Component level	Yes	Yes (line plots, histograms)	Yes	Yes	No	No
Welton [8]	2009	No	No	No	No	Yes	No	No	Yes (Model fit)
Coventry [29]	2020	Yes	No	No	No	No	No	No	No
Petropoulou [30]	2020	Yes	Intervention level	No	No	No	No	No	No
Smith [31]	2021	Yes	No	No	No	Yes	Yes	No	No
Sposito [32]	2021	No	Intervention level	No	No	No	No	No	No
Launder [33]	Unpublished	Yes	Component & Intervention level	No	No	No	No	No	No
Fujii [34]	2022	Yes	No	No	Yes (Funnel plot, league tables, risk of bias)	Yes	No	No	No
Dautzenberg [35]	2021	Yes	No	No	No	Yes	No	No	No
Dautzenberg [36]	2021	Yes	No	No	No	Yes	Yes	No	No
Burton [37]	2021	No	Component level	No	Yes (Risk of bias)	No	No	No	No
Wang [38]	2021	Yes	Intervention level	No	Yes (Funnel plot, risk of bias)	No	No	No	No
Fong [39]	2021	Yes	Component & Intervention level	No	Yes (Funnel plot, risk of bias)	No	No	No	No
Roberts [40]	2021	Yes	No	No	Yes (Risk of bias)	No	No	No	No
Cintra [41]	2021	No	Intervention level	No	No	No	Yes	No	No
Bálint [42]	2021	Yes	No	No	No	Yes	Yes	No	No
Veroniki [43]	2022	Yes	No	No	No	Yes	Yes	No	No

Across the 34 included papers, 26 included a network diagram [3, 11–13, 17–21, 23–27, 29–31, 33–36, 38–40, 42, 43], 17 included a summary forest plot at either the component or intervention level [1, 3, 9, 12, 13, 20, 21, 23, 27, 28, 30, 32, 33, 37–39, 41] and 16 included other plots [1, 3, 12, 17, 20, 22, 23, 25–28, 34, 37–40] (Table 1). One paper included no plots [8]. Nineteen papers included tables reporting component effects [1, 3, 8, 9, 11, 13, 17–19, 22, 24, 25, 28, 31, 34–36, 42, 43], eleven reported intervention effects (which could include multiple components) [13, 18, 19, 24, 26, 28, 31, 36, 41–43], two reported ranking of components [12, 13] and nine used tables to report other information [3, 8, 11, 12, 18, 20, 22, 23, 25]. Ten papers included no tables [21, 27, 29, 30, 32, 33, 37–40].

We describe in further detail below approaches for visualising availability of data/data structure. In the Supplementary Material, we consider in detail plots specifically for reporting single outcomes, plots for multiple outcomes and/or multiple models and information presented in tables. Where appropriate, we include the relevant cross reference to the graph vignettes developed by Kossemier et al*.* [15]. A brief summary of the review in the Supplementary material is provided below, and this is followed by a brief statement regarding how the review informed and motivated the development of the novel plots presented in Novel plots Section.

#### Availability of data/data structure

Across all thirty-four papers, we only identified one plot type – network diagram (Graph 11.1, supplementary appendix of [15])—used to visualise the data structure included in the CNMA. Twenty-six articles (76%) included a network diagram. In all cases, where studies considered more than one outcome, multiple network plots were presented – one for each outcome e.g. Freeman et al. [3].

Between reviews, network diagrams varied in a number of ways including size of component/intervention nodes, thickness of lines connecting components/interventions and use of colour. In some studies, component/intervention nodes were the same size for all components/interventions whereas in other studies the size of the nodes was weighted based on the number of participants/trials including each component/intervention. Similarly, in some studies the thickness of the lines connecting components/interventions was identical for all lines in the plot whereas in other studies the thickness of the lines was weighted based on the number of trials comparing each combination of components/interventions. In most cases, these two characteristics were implemented together. Some plots were all the same colour whereas in other plots the component/intervention nodes were a different colour to the lines connecting the components/interventions representing the presence of direct evidence comparing the components/interventions.

Following publication of the PRISMA-NMA guidelines in 2015 [44], the presentation of network diagrams in standard NMA papers is now routine practice and it is therefore perhaps not surprising that the majority of CNMA papers in our review included a network diagram. One consideration with network diagrams is the size of the network. Network diagrams can be used for large numbers of interventions, e.g. Fig. 1 from [17] includes 51 interventions, but there is still an upper limit. For example, a network diagram for the CNMA reported in [1] was not included in the publication because with 38 components and 437 unique combinations of components the diagram was not legible.

The data for a CNMA is not always included in a paper or its supplementary material. In this case, network diagrams can be a useful tool for describing the available data. Network diagrams can give an indication of the number of studies, interventions, components or participants included in the data. They can highlight which pairwise comparisons are informed by direct evidence only, indirect evidence only or a combination of both direct and indirect evidence. Importantly for CNMA, they may also give an indication of which components are commonly given together in combination with other components. NMA requires a connected network, an assumption which a network diagram can verify. However, a disconnected network, in which many interventions have components in common, may still be suitable for analysis as a CNMA [12].

Additional approaches to visualising the availability of data included a heat map plot indicating the number of trial arms including pairwise combinations of components [1] and a forest plot to present the observed effect sizes from individual trials [3]. The heat map plot is explored in more detail in the novel plots section. The forest plot in [3] has been organised by increasing number of components or intervention complexity. If all the components in an intervention have a beneficial/negative effect then this approach based on observed effect sizes may hint at whether there is a relationship between the number of components in an intervention and the effect size prior to any CNMA models being fitted. However, there is no obvious place to position multi-arm trials so these have been placed at the bottom of the forest plot. This approach will also be of limited use when many interventions are compared head-to-head rather than to a common comparator, such as usual care.

#### Summary of results reporting

For CNMA, effect sizes and uncertainty were most commonly presented as summary forest plots whilst ranking of components and interventions was commonly presented using cumulative ranking plots (Graph 11.7, supplementary appendix of [15]). These two types of plots, which are commonly used for standard meta-analysis and NMA, can be easily adapted for better use in a CNMA. Consideration of the results presented in tables showed that the majority of tables could have been replaced with a summary forest plot or ranking plot.

#### Conclusions of review informing the development of novel plots

As Summary of results reporting Section summarises, existing meta-analysis plot types are readily adaptable to report the results of CNMA. However, the current approach of using a network diagram(s) to visualise the data structure and availability of data is limited for CNMA for the reasons discussed in Availability of data/data structure Section. In Novel plots Section we consider three novel plots for visualising the data structure and/or the availability of data for a CNMA; two novel in their application and one novel in design.

## Novel plots

In this section, we present plot types not previously used in published CNMA and a plot we developed specifically for CNMA to visualise the data structure and/or availability of data. These plots are presented for our motivating dataset of psychological preparations prior to surgery [3]. This dataset includes six components making up 19 unique interventions across 36 trials (30 two-arm, 4 three-arm and 2 four-arm trials) for the outcome length of stay in hospital.

### CNMA-UpSet plot

**Fig. 2:**
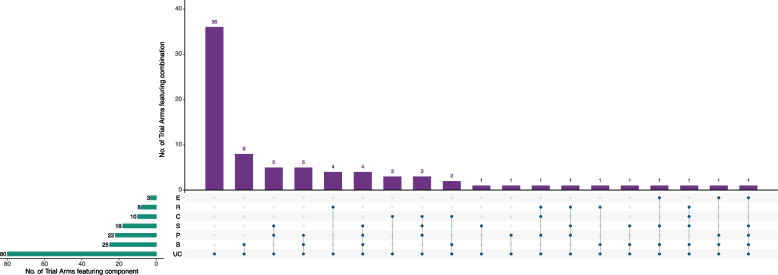
CNMA-UpSet plot for the psychological preparations dataset. In the matrix of dots in the bottom-right hand corner each row represents a component and each column represents an intervention. The solid vertical lines connecting dots within a column indicate which components are included in each intervention. If an intervention consists of a single component there is no vertical line. Above the matrix of dots, a vertical bar chart represents the number of trial arms for each unique intervention. To the left of the matrix of dots, a horizontal bar chart represents the number of trial arms including each specific component (regardless of whether it was included in isolation or in combination with other components). E = emotion-focused techniques, R = relaxation, C = cognitive interventions, S = sensory information, P = procedural information, B = behavioural instruction, UC = Usual care

The UpSet plot was initially designed as an alternative to the Venn diagram to enable visualisation of sets and their intersections [45]. Whilst a Venn diagram is restricted to around four sets, the matrix form of the UpSet plot allows it to be used to visualise many more sets and their intersections. In the CNMA setting, each unique combination of intervention components is considered to be an intersection. The CNMA-UpSet plot consists of three elements (see Fig. 2 for an example CNMA-UpSet plot of 19 interventions (including usual care)):

- The bottom right-hand corner contains a matrix of dots in which each row represents a component and each column represents an intervention containing a number of coloured dots to denote whether the component in the row is present in the intervention. The solid vertical lines connecting dots within a column indicate which components are included in each intervention. Interventions where no solid vertical line is present correspond to interventions in which a single component was administered.
- Above the matrix of dots, a vertical bar chart represents the number of trial arms for each unique intervention.
- To the left of the matrix of dots, a horizontal bar chart represents the number of trial arms including each specific component (regardless of whether it was included in isolation or in combination with other components).

UpSet plots can be created in R using the package *UpSetR* [46]. R code to re-create Fig. 2 is available in the Supplementary Material.

The most popular approach to date for visualising the data structure for a CNMA is the network plot, initially designed for NMA. A CNMA-UpSet plot can be created for a greater number of unique combinations of components than a network plot so it scales well to larger networks. For example, although not included in the final publication, a CNMA-UpSet plot for all possible combinations of components which occurred in at least 3 arms was created to explore the data structure for a large CNMA of behavioural interventions for smoking cessation consisting of 284 trials, 659 trial arms and 38 components [1].

A network diagram can show the number of trials directly comparing two interventions, which is informative for NMA and can be fitted using contrast-based data but requires a connected network (at the intervention level). Compared to a network diagram, the CNMA-UpSet plot can identify the number of trial arms featuring each component as well as the number of trial arms featuring each unique combination of components, which are more informative for CNMA. It is fitted using arm-based data and does not necessarily require a connected network (at the intervention level). Both a network diagram and the CNMA-UpSet plot can show the number of unique combinations of components present in a dataset.

### CNMA heat map

Another existing plot type, which can be useful to explore the structure of a CNMA dataset, is a heat map plot. A CNMA heat map can be created to visualise:

1. The correlation indicating how often two components are given together. A correlation of 1 indicates that the two components are always given together whilst a correlation of 0 indicates that the two components are never given together.
2. The number of trial arms in which two components are both included.

A CNMA heat map provides a grid in which all components are listed along both the x-axis and the y-axis. The cells in the grid are then coloured to represent either the correlation between the components listed on the corresponding row and column, or, the number of trial arms in which those two components are given together. Such information is valuable when fitting CNMA models as it highlights collinearity between components (including identifying if there are any pairs of components which are always given together) which may cause problems of parameter identifiability and thus greatly inflated standard errors or total failure to estimate component effects. Importantly, this information cannot be identified from a standard network diagram. Furthermore, the size of the heat map depends on the number of components within a dataset and can therefore scale up well to large networks.

In some cases, the additive effects assumption for CNMA may not be appropriate and it may be important to consider pairwise interactions between components. In this situation, a heat map can be utilised to aid decisions about which pairwise interactions would be feasible to include in a CNMA model by identifying the number of data points for each pairwise combination of components that have been trialled together.

**Fig. 3:**
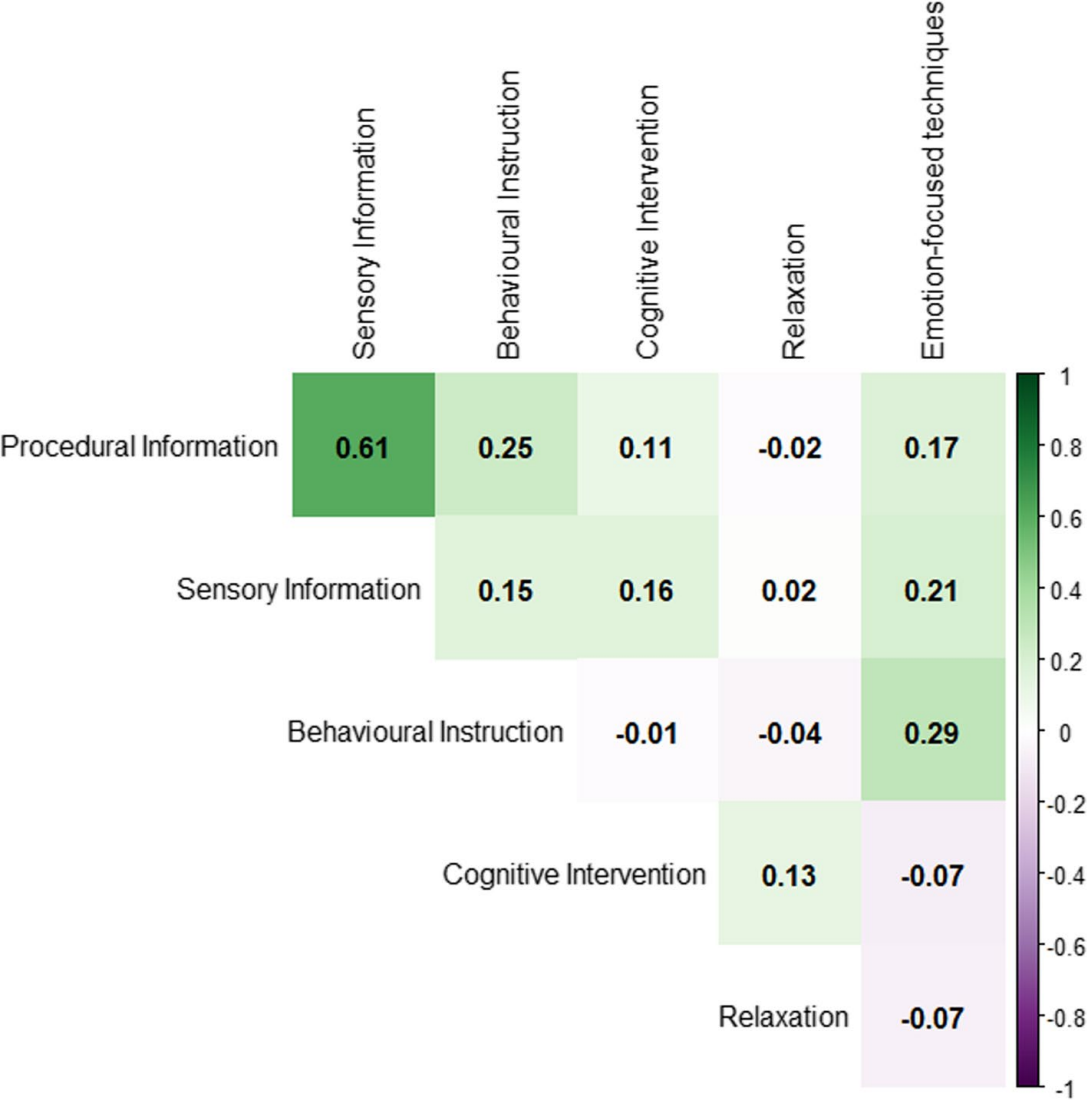
CNMA heat map presenting correlations between two components for the psychological preparations dataset

**Fig. 4:**
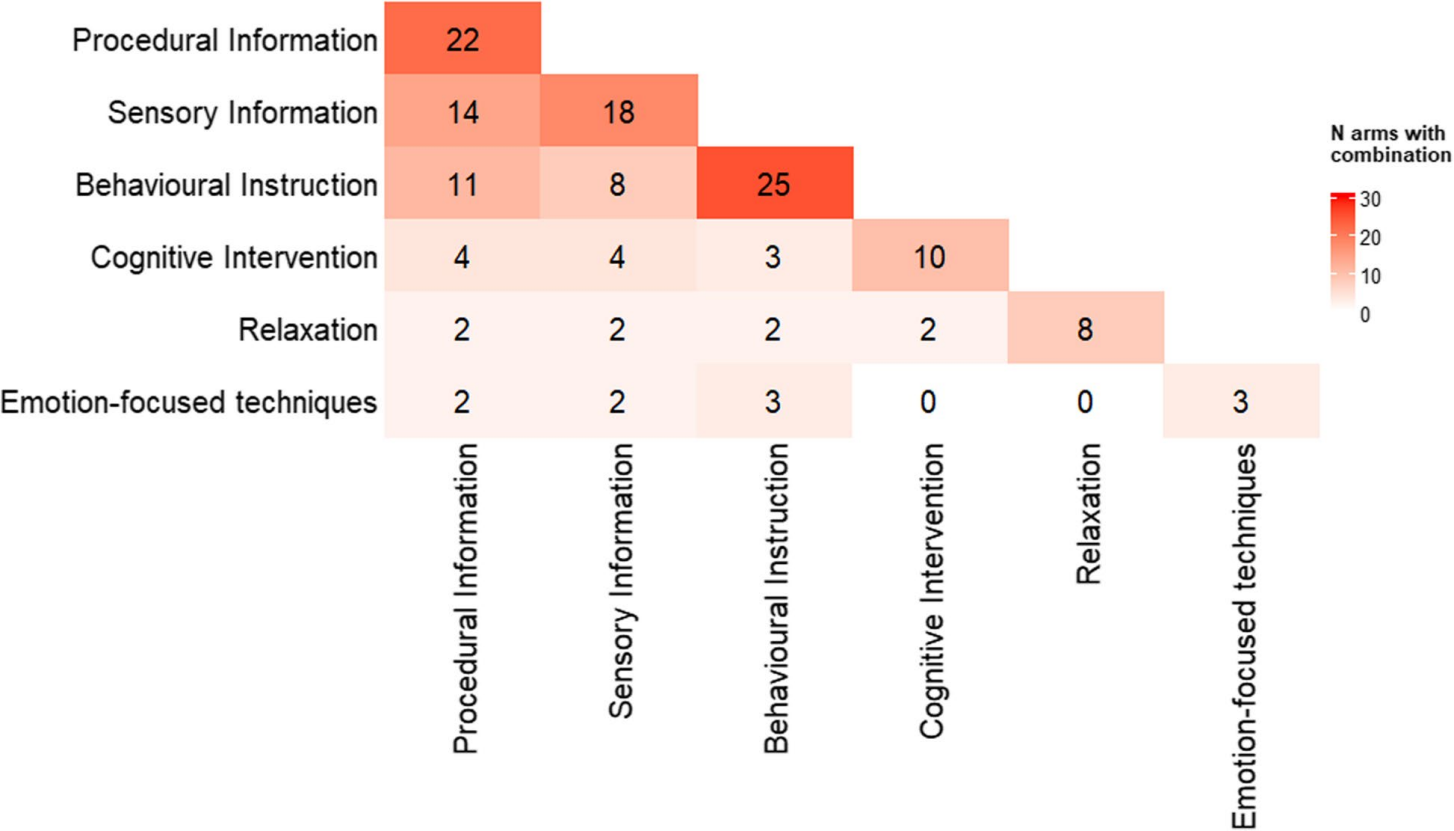
CNMA heat map showing frequency and combinations of components across study arms for the psychological preparations dataset. The numbers indicate the number of study arms that contained the components in the corresponding row and column. For cells where the row and column component are the same, the frequency of that component is shown

An example of a CNMA heat map presenting correlations between two components is given in Fig. 3. In Fig. 3, white represents no correlation, green represents positive correlation and purple represents negative correlation. The stronger the green or purple colour, the stronger the positive or negative correlation. An example showing the frequency and combinations of components across study arms is given in Fig. 4. In Fig. 4, white represents low frequencies whilst red represents high frequencies. The stronger the red colour the higher the frequency. Figure 3 was created in R using the package *corrplot* [47] and Fig. 4 was created in R using the package *ComplexHeatmap* [48]. R code to re-create these plots is given in the Supplementary Material.

### CNMA-circle plot

**Fig. 5:**
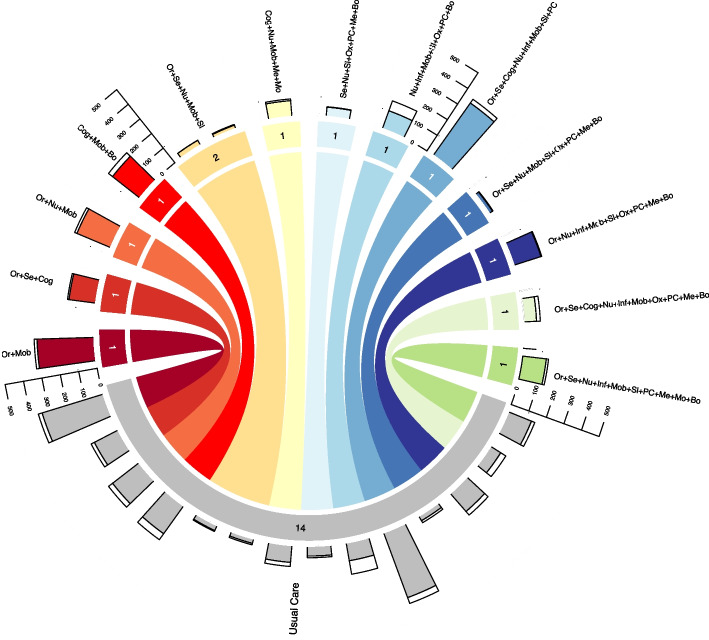
CNMA-circle plot with composite bar chart for the delirium prevention dataset. Links between interventions are coloured by the difference between arms. Links are coloured grey when the combination of components that differ between arms are not trialled in any single arm in the network. Multi-arm trials are denoted by multiple thinner connecting links. Numbers at the end of each link represent the number of trial arms for each intervention. The coloured section of the bars represents the number of patients without delirium and the white section the number of patients with delirium. In this network, all studies compared interventions to usual care so there are no head-to-head comparisons between interventions. Or = Re-orientation & familiar objects, Se = Attention to sensory deprivation, Cog = Cognitive stimulation, Nu = Nutrition & hydration, Inf = Identification of infection, Mob = Mobilisation, Sl = Sleep hygiene, Ox = Oxygenation, PC = Pain control, Me = Medication review, Mo = Mood, Bo = Bowel & bladder care

The CNMA-circle plot is a circos-type plot [49] (Graph 9.10, supplementary appendix of [15]) developed to visualise the data structure for an additive effects CNMA model and allows presentation of multiple dimensions of information. Prior to presenting the plot for the psychological preparations prior to surgery dataset, an example presenting a relatively small and simple CNMA data structure is initially presented in Fig. 5 for data from a published CNMA of Non-pharmacological interventions for preventing delirium in hospitalized patients [37].

All interventions (i.e., unique combinations of components, including singular components) included in at least one trial arm are plotted in segments around the circle (using a unique colour). The width of each segment represents the number of trial arms that tested each intervention and the ordering of segments is determined by the number of components they include (increasing in a clockwise direction from usual care). Links are plotted between segments where trials have compared the respective interventions; for example, in Fig. 5 there are 13 different unique combinations of components (interventions) trialled, and each trial has compared one of the component combinations to usual care, which is indicated by all links connecting with the usual care segment. All but one component combination (excluding usual care) is unique, with Or + Se + Nu + Mob + Sl being compared to usual care in 2 trials (indicated by the faun coloured link being thicker than the others). The colours of these *links* represent the combination of components that *differ* between the connected interventions. In Fig. 5, each intervention is compared to usual care (which is assumed common across each trial arm of every trial) thus the colour of the links is always the same as that of the combination being trialled against usual care. Finally, a stacked bar chart is included around the edge of the plot. There is one bar per study arm and they provide the data for the (binary) delirium outcome for the study, with the coloured section indicating the number of patients without delirium and the white section indicating the number who did get delirium.

**Fig. 6:**
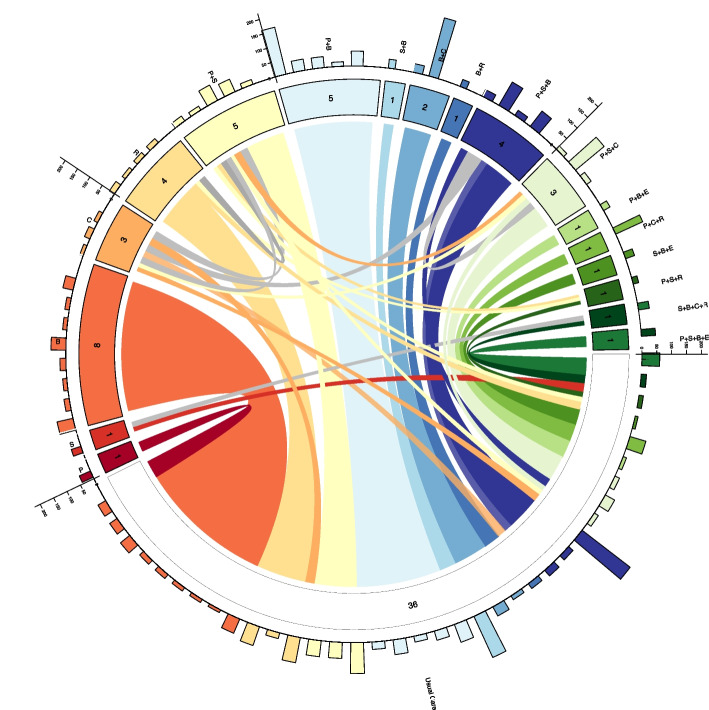
CNMA-circle plot for the psychological preparations dataset. Links between interventions are coloured by the difference between arms. Links are coloured grey when the combination of components that differ between arms are not trialled in any single arm in the network. Multi-arm trials are denoted by multiple thinner connecting links. Numbers at the end of each link represent the number of trial arms for each intervention. Bars represent the sample size for each trial arm. In this network all studies included usual care. For trial arms including additional components, usual care has been excluded from the intervention label. E = emotion-focused techniques, R = relaxation, C = cognitive interventions, S = sensory information, P = procedural information, B = behavioural instruction

The data structure for the psychological preparations dataset is presented as a CNMA-circle plot in Fig. 6. This plot is more complicated than for the delirium example reflecting the more complicated data structure. The first new feature to note is the colouring of links where comparisons are made between regimes where both include one or more components. For example, the link for the trial comparing relaxation (denoted R) against procedural information, sensory information and relaxation (denoted P + S + R) is given the colour designated for procedural information and sensory information (denoted P + S) since this is the component combination *difference* between arms and is appropriately coloured pale yellow (the colour of the P + S segment). Links are coloured grey when the combination of components that differ between arms are not trialled in any single arm in the network. For example, the comparison of C with P + S (which has a difference of C – P – S which isn’t (and cannot be) represented in any trial arm and is distinct from P + S + C) is coloured grey. This dataset also includes multi-arm trials, and these are denoted by multiple (thinner) connecting links of different colours leading from each of the trial arms. For example, Fig. 6 includes a 3-arm trial comparing usual care with sensory information (denoted S), and the combination sensory information, behavioural instruction, cognitive interventions and relaxation (denoted S + B + C + R); this is depicted by a red link connecting usual care and S, grey link connecting S and S + B + C + R, and dark green link connecting S + B + C + R and usual care (also shown separately in Figure S1 for clarity). As before, a circular bar chart is placed around the edge of the plot; as each bar represents a trial arm, the reader can ascertain the number of arms for each intervention (the number is also given on the colour indicating segment for each unique intervention). Since the primary outcome in this dataset is continuous and not binary (as before), it is not possible to represent the outcome data via a stacked bar chart, therefore a simple bar is presented indicating the sample size for each trial arm (Fig. 6). The CNMA-circle plot was created using the R package *circlize* [50] and R code to re-create Figs. 5 and 6 is available in the Supplementary Material.

The CNMA-circle plot has some similarities with the network plot and the CNMA-Upset plot. All three plots display the interventions present in the network and the number of trial arms for each intervention. However, the CNMA-circle plot is the only plot which, in the presence of trials without a usual care arm, visualises the combination of components that differ between trial arms. In conjunction with the CNMA-heat map this may help identify which components can be estimated from an additive effects model and inform any decisions around modelling interactions between components.

The CNMA-circle plot can be scaled up to networks larger than our illustrative examples. However, similarly to a network plot, the size of which is often limited by the number of interventions, the size of the CNMA-circle plot will be limited by the number of unique interventions.

The CNMA-circle plot includes an outer bar chart which, in this paper, represents the number of patients in each trial and, additionally, for binary outcomes the proportion of patients who experienced the outcome of interest. However, this could be customised to display alternative information, such as the proportion of patients at each level of a categorical covariate. Alternatively, the bar chart could be replaced with a box plot to illustrate the distribution of a continuous outcome within each trial arm.

## Discussion

This paper reviewed the presentational displays currently used for reporting CNMAs. It found that, whilst summary forest plots and ranking plots were successfully transferred from NMA to CNMA reporting, network diagrams provided limited information regarding the complex data structures associated with CNMA. To address this issue, this paper presented three novel approaches to presenting complex data structures for CNMA. Visualisation of the available data structures for CNMA is important to help inform and guide model choice.

The CNMA-UpSet plot presents arm-level data and is suitable for networks with large numbers of components or combinations of components. Heat maps can be utilised to inform decisions about which pairwise interactions to consider for inclusion in a CNMA model. The CNMA-circle plot visualises the combinations of components which differ between trial arms and offers flexibility in presenting additional information such as the number of patients experiencing the outcome of interest in each arm. The CNMA-Upset plot and the CNMA-circle plot are designed to display multiple dimensions of information simultaneously to enhance understanding of the CNMA evidence base including number of trial arms featuring each component or combination of components, and number of patients in each trial arm. The CNMA-circle plot also clearly shows where direct head-to-head evidence is available and where comparisons will be estimated based on the CNMA model predictions only. To complement these two visualisations, the proposed CNMA heat map presents a correlation plot which highlights the collinearity between components and enables pairs of components that are always given in combination to be identified (which would lead to a necessary re-defining of the component definitions before a CNMA model could be fit successfully); this plot may be particularly useful for CNMA models that consider pairwise interactions thus relaxing the additive assumption. The circle plot is most limited in terms of scalability followed by the UpSet plot. However, all three visualisations have good scalability properties in terms of number of studies, interventions and components. Finally, development of the three novel plots was partially limited to availability of R packages and their own drawing restrictions; in spite of this, we do not feel that the restrictions limit their potential to improve understanding of complex CNMA structures.

The outer bar chart of the CNMA-circle plot can be customised in a number of ways and can display either covariate or outcome data. The downside to having a plot which allows a variety of “mix-and-match” options is that the R code to create the CNMA-circle plot is complex. Although we provide code for the two circle plots presented in this paper, it would be desirable to generalise this code by building a wrapper package around *circlize*—specifically for building CNMA-circle plots—to increase the accessibility of this new plot. To further aid understanding of the data structure, interactivity could be added to the CNMA-circle plot so that certain features could be highlighted, for example, all trial arms using components X and Y, at the click of a button, or alternative information displayed around the edge of the plot and this is ongoing work.

One limitation of our review is that we conducted a citation search of two data sources rather than a systematic literature review. The authors of this paper are aware that some papers which include CNMA (and were eligible for inclusion in our review) do not include CNMA in the title, instead using NMA. Therefore, a citation review of two key papers was chosen as a pragmatic way to identify papers which applied CNMA methodology whilst avoiding the need to screen thousands of NMA articles which were not relevant to our review. However, it is possible that our approach may have missed some CNMAs and there is the possibility that these missed papers used visualisation approaches that were not identified in this paper. Throughout this paper we have assumed components are treated as binary variables – they are either present in an intervention or not. However, it is possible that components could be treated as continuous variables representing the intensity of a component within an intervention. In this case, to create the plots in this paper categorisation would be required.

As we were completing the work presented here, two papers considering visualisations for syntheses of multicomponent intervention studies were published. One paper focused specifically on visualising the evidence structure within a CNMA and proposed the use of signal-flow graphs [14]. As previously described, in some cases not all components in a CNMA can be uniquely estimated and Li et al. show how a signal-flow graph can be used to identify which components can be uniquely estimated. The signal-flow graph does not include information such as the number of trial arms including each specific component, the number of trial arms including each unique intervention, how often two components are given together. Therefore, we believe the signal-flow graph complements the plots presented in this paper and together they can both help inform model choice.

Another paper considered both the data structure and results of analysis [51]. However, this paper focuses on the analysis of multicomponent studies using a (standard) NMA model whereas we focused our review on CNMA analyses and aimed to develop plots for this context. The only overlap in visualisations presented between the two papers is the use of a heat map type plot for displaying the frequency of pairs of components used together. We believe the two papers complement each other offering a broad suite of visualisations to use across both NMA and CNMA analyses for multicomponent interventions. Importantly, when conducting a synthesis of such studies, it may not be obvious which model is most appropriate, and an analyst may consider using NMA and CNMA models (the latter with and without interaction effects) [8]. Given the plots in this paper consider the data structure (and not analysis results), they are relevant and appropriate even if a classic NMA model is ultimately used in the final analysis. Similarly, the plots presented by Seitidis et al. [51] may be helpful to the analyst in deciding between the use of NMA and CNMA models.

The visualisations developed in this paper all focus on the presentation of a single outcome. The UpSet plot and heat map could be used to describe all included studies in a systematic review, regardless of outcome. However, the feasibility of adapting all three plots to display the data structure for multiple outcomes simultaneously forms a topic for future research. Future research could also consider incorporating quality assessments (e.g. risk of bias or GRADE (Grading of Recommendations Assessment, Development, and Evaluations)) in visualisations.

To conclude, as CNMA becomes more widely used for the evaluation of multiple component interventions, including combinations of components not previously trialled, we believe that the novel CNMA-specific multi-dimensional visualisations developed in this paper will be important to aid understanding of the complex data structure by analysts and end-users, and facilitate interpretation of the analysis results.

### Supplementary Information

**Additional file 1.** Supplementary material.

## Data Availability

The psychological preparation dataset is available at: https://zenodo.org/record/8006703 The delirium prevention dataset is available at: https://doi.org/10.1002/14651858.CD013307.pub2 R code to produce the plots in this paper is provided in the Supplementary Material.
