# Supplementary material for: Data visualisation approaches for component network meta-analysis: visualising the data structure
Source: BMC Med Res Methodol. 2023 Sep 15;23:208. doi: 10.1186/s12874-023-02026-z (PMC10502971; doi:10.1186/s12874-023-02026-z)
Supplement: Supplementary file 1 — Additional file 1. Supplementary material. [file 12874_2023_2026_MOESM1_ESM.docx]

**Graphical approaches for component network meta-analysis**

**Supplementary Material**

**Additional information from the review of visualisations identified**

We describe in further detail the plots for reporting single outcomes, plots for multiple outcomes and/or multiple models and information presented in tables. Where appropriate, we include either the relevant cross reference to the graph vignettes developed by Kossmeier *et al.* (1).

**Single outcome**

*Visualising effect size and uncertainty*

Twelve studies used a summary forest plot (Graph 11.2, supplementary appendix of (1)) to visualise either intervention or component effect sizes and uncertainty for a single outcome (2–13). Summary forest plots can be customised through the use of colour for the inclusion of additional details such as number of trials, number of trial arms, number of patients, number of events, and prediction intervals if applicable.

Two studies (12,14) which both considered the impact of a continuous covariate on the outcome of interest used meta-regression and line graphs to show how the outcome changes across different values of the continuous covariate. In study (14), all components were plotted on the same set of axes with colour used to distinguish between components (Graph 11.8, supplementary appendix of (1)). In study (12), each component was given a different set of axes, a line was added for the outcome for patients who did not receive any intervention and colour used to add an additional line for the effect of the component in patients who did receive the component of interest. Where appropriate, the best approach may be to combine these two approaches by plotting all the components on the same axes and including a line for patients receiving usual care. This would allow easy comparison between components as well as visualising the effect size for each component compared to usual care.

One study (15) displayed histograms of the posterior distribution for each component in the network alongside a line of null effect. This approach using density plots provides little additional information beyond the point estimate and credible interval, which could be presented in a summary forest plot. Arguably, presenting each component as a separate density plot makes it harder to visually compare the effect of each component to the other components in the network. If density plots are to be used we would recommend plotting the distributions on the same plot although we acknowledge that this approach is likely to be only applicable to networks with smaller numbers of components. Additionally the density plots include a probability statement of how likely it was for the hazard ratio for each component to be less than one, which may be useful as it gives a direct probability statement about the likelihood of a beneficial effect but (based on the authors experiences) is rarely seen, even for standard NMA (but does not need to be linked to a density plot of the posterior distribution).

*Visualising ranking of components/interventions*

Only three studies (9,12,14) used a graphical approach to visualise the ranking of components. One study (12) presented the probability of each component having each rank as a series of line graphs, one per component (Graph 11.7, supplementary appendix of (1)). For smaller networks, comparison between components may be improved by placing all the component’s profiles on the same set of axes and using colour to distinguish between the components. Study (9) used a similar approach when considering multiple outcomes which is discussed further below.

In the presence of a categorical covariate, one study (14) presented the probability of each component being the best component, conditional on each category of the covariate, using a bar chart. Colour was used to aid visual comparison of the same component across different levels of the covariate.

One study (9) calculated both the surface under the cumulative ranking (SUCRA) curve and P-score for each component allowing them to rank the components for a single outcome (risk of adverse effects) and presented these results as a scatter plot with SUCRA on the x-axis and P-score on the y-axis. SUCRA is a single numerical summary proposed to supplement the graphical display of cumulative probability plots (16). A component, which always ranks first will have a SUCRA value of 1 and a component which always ranks last will have a SUCRA value of 0 (16). Thus, SUCRA describes the probability of a component being identified as the most effective component. A P-score is a frequentist analogue to the Bayesian SUCRA, which measures the certainty that a component is more effective than the other components (17). Colour was used to label the combinations of components with white representing low SUCRA/P-score and red representing high SUCRA/P-score. Due to their definitions, SUCRA and P-scores are typically highly correlated. In this case, the scatter plot highlights one particular component for which there is some discrepancy between the SUCRA and P-score ranking.

Whilst not quantifying the ranking of components, one additional study (2) ordered the summary forest plot of intervention effect sizes and their 95% credible intervals so that, within each component category, the component with the highest odds ratio was at the top and the component with the lowest odds ratio was at the bottom. This allows the reader to identify whether there is any overlap between the effect estimates and could be more informative than a ranking score that does not take into account the uncertainty around an effect estimate or the quality of the evidence and could therefore be misleading (18).

**Multiple outcomes and/or multiple models**

*Visualising effect size and uncertainty*

Five studies (14,19–22) reported results from multiple outcomes using summary forest plots but presented a separate summary forest plot for each outcome. Five studies (4,6,9,20,21) reported results from multiple CNMA models on the same summary forest plot, typically these models were the additive effects CNMA, and the interaction CNMA or classic NMA. All summary forest plots were produced using the R netmeta package. All five studies used colour to distinguish between the different models. When reporting multiple models, displaying the results within a single visualisation facilitates the comparison of the results between models and can help identify whether the results remain consistent across different model assumptions. When reporting multiple outcomes, it may also be desirable to place them on the same plot to determine whether the most effective components/interventions are consistent across the multiple outcomes. However, this may prove challenging if the outcomes are of different types (e.g. one binary and one continuous outcome) or, in the case of continuous outcomes, the effect scales take different values, in which case, it may make sense to report the results using different plots.

One study (23) used a modification of the summary forest plot to present the results for a single outcome from multiple models. They used a caterpillar plot in which the models were presented on the x-axis and the effect sizes on the y-axis so that the lines, representing the mean and 95% credible intervals for the effects of interventions, run vertically rather than horizontally.

One study (8) compared the effect sizes for combinations of components (interventions) from nine different models using a line graph in which the x-axis distinguishes models and the y-axis indicates the intervention effect estimate. Each intervention in the network was represented using a different colour line. An advantage to this approach is that the reader can easily visualise how the intervention effect estimates vary across the nine different models for each intervention.

Two studies (9,24) used two different formats of a league table in which the intervention labels (i.e. single components and combinations of components) are placed along the leading diagonal and effect sizes below the leading diagonal represent one outcome measure and above the leading diagonal a second outcome. In one of the studies (9), the table was coloured based on statistical significance across a number of different models. Despite having a table format, the introduction of colour allows readers to identify patterns and easily identify the most effective interventions. In the other study (24) prediction intervals were also included, and those not including the null effect were highlighted in bold.

The work in a PhD dissertation (12) displayed the posterior distributions from two models for each component by plotting a histogram for each component and using colour to distinguish between models. It is possible that the components could have been placed on the same set of axes to allow easier comparison between components. However, it would not take many components or models before the plot would become crowded and difficult to distinguish the different lines.

*Visualising ranking of components/interventions*

One study (14) presented the probability of each component obtaining each rank for three outcomes by plotting a separate line graph for each component and using colour to distinguish between the three outcomes.

Another study (9) presented the cumulative probability rank curves for single and combinations of components. The colour of the line representing each component or combination of components was coloured based on the SUCRA value with low SUCRA values represented by yellow and high SUCRA values represented by dark blue. The SUCRA value for each single or combination of components was displayed alongside a label in the figure legend with labels ordered based on the SUCRA value. This plot included 29 single or combinations of components, and, whilst it is hard to determine from the lines themselves which component is best, the ordering of the figure legend facilitates this. Each outcome was presented on a separate plot. The same study also presented the median ranks and associated 95% credible intervals for two outcomes as a summary forest plot (9). The plot also contained SUCRA values. Colour is not used to distinguish between the two outcomes but rather colour is used for background shading to enable the reader to easily distinguish each component. With 29 single or combinations of components it is easier to read this plot than the cumulative rank plot.

The same study (9) also presented a scatter plot of the SUCRA values for the two outcomes (Graph 11.17, supplementary appendix of (1)) and a separate scatter plot of the P-scores for the two outcomes. The SUCRA scatter plot used colour to label the combinations of components with white representing low SUCRA and dark blue representing high SUCRA. In the P-score plot, solid lines are used to connect interventions to show superiority or inferiority of interventions to one another on two outcomes simultaneously (Graph 11.22, supplementary appendix of (1)). Dashed lines are used to help readers read off values form both axes. Scatter plots are a good option when looking to display the results from two outcomes or two models. However, when the number of outcomes or models increases line plots are more relevant.

**Other plots**

In our review, we also identified three other types of plot: risk of bias plots, funnel plots and model fit plots. Twelve papers (2,5,9,11,13,15,20,21,24–27) included a risk of bias plot, either the summary stacked horizontal bar chart or risk of bias for each domain for each study using the standard Cochrane risk of bias plots. One study (13) displayed the risk of bias information alongside effect sizes from a pairwise meta-analysis but none of the studies used visualisations to display risk of bias information alongside effect sizes from a CNMA. Doing so could aid interpretation of the results and help identify any comparisons particularly influenced by high levels of potential bias.

Six studies reported funnel plots. The purpose of a funnel plot is to identify evidence of small-study effects (often termed publication bias) and ideally, the plot will look symmetrical (Graph 11.10, supplementary appendix of (1)). Two studies (23,28) considered funnel plots for usual care versus any intervention. One study (9) used colour whilst three studies (20,21,24) used different plotting symbols to distinguish between studies reporting different comparisons of interventions. Both of these approaches have drawbacks. The first approach is only relevant if there is a natural way of identifying a control and an experimental intervention for each trial, which may not always be the case. The second approach may not be appropriate if there are more unique comparisons of combinations of components or interventions than distinguishable sets of colours or symbols.

To assess model fit, one study (2) plotted leverage on the y-axis, deviance residuals on the x-axis and added contour lines to represent different values of the deviance information criteria (DIC). Each trial arm contributed one point to the plot and points were colour coded based on the risk of bias assessment. The colour coding showed that, generally, the study arms with poor fit were from studies with high risk of bias. Although in this example risk of bias was considered alongside model fit, it does hint at the potential advantages of considering risk of bias directly alongside estimates of effect size.

**Tables**

Twenty-four studies chose to present some results using tables. A list of tables is included in Table S1. One paper included all tables and no figures (29). Across 24 studies, we identified 52 tables reporting CNMA results.

Thirty-six tables presented relative effect sizes. Twelve of these tables included additional details beyond the mean/median effect size and 95% confidence/credibility intervals. Most commonly, this included number of patients, number of study arms, number of studies, heterogeneity statistics or combinations of these items. Inclusion of additional data on the number of study arms including each component gives a measure of how often the component is used in the studies included in the CNMA (i.e. the amount of direct evidence available to inform the component effect sizes). Other items included within tables of effect sizes included absolute effect sizes, probability of being the best, p-values and model fit statistics (e.g. deviance information criteria). Sixteen (of 36) tables presented effect sizes for a single outcome from a single model. The remaining studies presented effect sizes for either multiple outcomes, multiple models or both.

Tables often allow for easy comparison between components/outcomes/models by placing information in a column or row. Study (14), table 1 provides an example of this. However, this table could also have been presented as a summary forest plot. Both relative and absolute component or intervention effects could be displayed on summary forest plots. Summary forest plots can be modified to include number of patients, number of study arms, number of studies, probability best or combinations of these items. Study (15), table 3 is an exemplary example of a relatively compact table that manages to communicate a lot of information. The table contains the effect sizes, 95% credible intervals and probability best for five components across seven outcomes.

Two studies (9,28) reported risk of bias in tables, but as previously described, these are standard figures available for presenting this information for pairwise meta-analysis and classic NMA. Five studies (3,8,9,28,29) reported ranking information in tables which could have been presented more accessibly using one of the plots for ranking components discussed above.

Six studies (5,14,15,26,29,30) reported model fit statistics such as the deviance information criteria (DIC) in tables. With DIC interest lies in the difference between DIC values from different models rather than absolute sizes and so we feel there is no benefit from graphically presenting this information.

One study ((9), supplemental table S3) reports effect sizes, credible intervals, between-study standard deviation, I-squared and DIC for covariates from a series of meta-regression models. We believe the effect size of the covariates is most relevant when presented alongside the component effects. For the continuous outcomes, this study could have used the line graph approach discussed earlier for continuous covariates.

**Table S1 List of Tables**

| **First Author** | **Publication Year** | **Table Number** | **Single or Multiple Outcome** | **Single or Multiple Model** | **What information is included in the table?** | **Alternative plot suggestion** |
| --- | --- | --- | --- | --- | --- | --- |
| Rücker (4) | 2019 | 2 | Single outcome | Multiple models | Effect sizes for 3 models | Summary forest plot |
| Rücker (8) | 2020 | 1 | Single outcome | Single model | P-scores | Any ranking plot |
| Rücker (8) | 2020 | 2 | Single outcome | Multiple models | Cochran's Q |  |
| Rücker (8) | 2020 | 3 | Single outcome | Multiple models | Cochran's Q |  |
| Mills (3) | 2011 | 2 | Single outcome | Single model | Effect size | Summary forest plot |
| Mills (3) | 2011 | 3 | Single outcome | Single model | Absolute treatment effect | Summary forest plot |
| Mills (3) | 2011 | 4 | Single outcome | Single model | Probability of each component taking each rank | Rank line plot |
| Pompoli (25) | 2018 | 3 | Multiple outcomes | Single model | Effect sizes plus no. of arms | Summary forest plot |
| Miklowitz (28) | 2020 | eTable 5B | N/A | N/A | Risk of bias | Risk of bias plot |
| Miklowitz (28) | 2020 | eTable 16 | Single outcome | Single model | SUCRA | Any ranking plot |
| Miklowitz (28) | 2020 | eTable 19 | Single outcome | Single model | Effect sizes | Summary forest plot |
| Mills (31) | 2012 | 2 | Single outcome | Multiple models | Effect sizes plus no. of trials and no. of patients | Summary forest plot |
| Mills (31) | 2012 | Web appendix 2 | Single outcome | Multiple models | Effect sizes plus no. of trials and no. of patients | Summary forest plot |
| Shi (9) | 2020 | 2 | N/A | N/A | Risk of bias | Risk of bias plot |
| Shi (9) | 2020 | 3 | Single outcome | Single model | Effect sizes plus standard deviation, I squared and DIC |  |
| Shi (9) | 2020 | 4 | Single outcome | Multiple models | SUCRA | Ranking plot for multiple models |
| Freeman (14) | 2018 | 1 | Multiple outcomes | Multiple models | Effect sizes | Summary forest plot |
| Freeman (14) | 2018 | 2 | Multiple outcomes | Single model | Effect sizes | Summary forest plot |
| Freeman (14) | 2018 | E.1 | Multiple outcomes | Multiple models | DIC |  |
| Freeman (14) | 2018 | E.2 | Single outcome | Single model | Effect sizes | Summary forest plot |
| Kabboul (15) | 2018 | 2 | Multiple outcomes | Multiple models | DIC |  |
| Kabboul (15) | 2018 | 3 | Multiple outcomes | Single model | Effect sizes and probability best |  |
| Kabboul (15) | 2018 | 4 | Single outcome | Single model | Effect sizes plus no. of arms | Summary forest plot |
| Hartmann-Boyce (2) | 2021 | 6 | Single outcome | Single model | Summary of findings - relative and absolute effect sizes plus no. of patients and certainty of evidence |  |
| López-López (5) | 2019 |  | Multiple outcomes | Multiple models | DIC |  |
| Melton (26) | 2020 | 3 | Single outcome | Single model | Effect size | Summary forest plot |
| Melton (26) | 2020 | 5 | Single outcome | Multiple models | DIC |  |
| Melton (26) | 2020 | 6 | Single outcome | Single model | Effect sizes | Summary forest plot |
| Madan (30) | 2014 | 3 | Single outcome | Multiple models | DIC |  |
| Madan (30) | 2014 | 4 | Multiple outcomes | Single model | Effect sizes | Summary forest plot |
| Madan (30) | 2014 | 5 | Multiple outcomes | Multiple models | Effect sizes | Summary forest plot |
| Chen (23) | 2012 | 25 | Single outcome | Multiple models | Effect sizes | Summary forest plot |
| Danko (12) | 2018 | 12 | Single outcome | Multiple models | Effect sizes | Summary forest plot |
| Danko (12) | 2018 | 13 | Single outcome | Single model | Effect sizes | Summary forest plot |
| Danko (12) | 2018 | 14 | Single outcome | Single model | Effect sizes | Summary forest plot |
| Welton (29) | 2009 | 3 | Multiple outcomes | Multiple models | DIC |  |
| Welton (29) | 2009 | 4 | Multiple outcomes | Multiple models | Effect sizes | Summary forest plot |
| Welton (29) | 2009 | 5 | Multiple outcomes | Single model | Probability best | Rank plot |
| Riemsma (32) | 2011 | 3.2 | Single outcome | Multiple models | Effect sizes | Summary forest plot |
| Riemsma (32) | 2011 | 3.3 | Single outcome | Multiple models | Effect sizes | Summary forest plot |
| Smith (33) | 2021 | 8 | Single outcome | Multiple models | Effect sizes plus between-study standard deviation | Summary forest plot |
| Fujii (24) | 2022 | 1 | Multiple outcomes | Multiple models | Effect sizes | Summary forest plot |
| Dautzenberg (34) | 2021 | S3 | Single outcome | Single model | Effect sizes | Summary forest plot |
| Dautzenberg (34) | 2021 | S4 | Single outcome | Single model | Effect sizes | Summary forest plot |
| Dautzenberg (35) | 2021 | S6 | Single outcome | Multiple models | Effect sizes | Summary forest plot |
| Dautzenberg (35) | 2021 | S7 | Single outcome | Single model | Effect sizes | Summary forest plot |
| Dautzenberg (35) | 2021 | S8 | Single outcome | Multiple models | Effect sizes | Summary forest plot |
| Dautzenberg (35) | 2021 | S9 | Single outcome | Single model | Effect sizes | Summary forest plot |
| Cintra (22) | 2021 | 1 | Single outcome | Single model | Effect sizes plus z-score & p-value | Summary forest plot |
| Cintra (22) | 2021 | 2 | Single outcome | Single model | Effect sizes plus z-score & p-value | Summary forest plot |
| Bálint (36) | 2021 | 2 | Multiple outcomes | Single model | Effect sizes plus p-value | Summary forest plot |
| Veroniki (37) | 2022 | 2 | Single outcome | Multiple models | Effect sizes plus between-study standard deviation and I^2^ | Summary forest plot |

DIC = Deviance information criterion, SUCRA = Surface under the cumulative Ranking curve


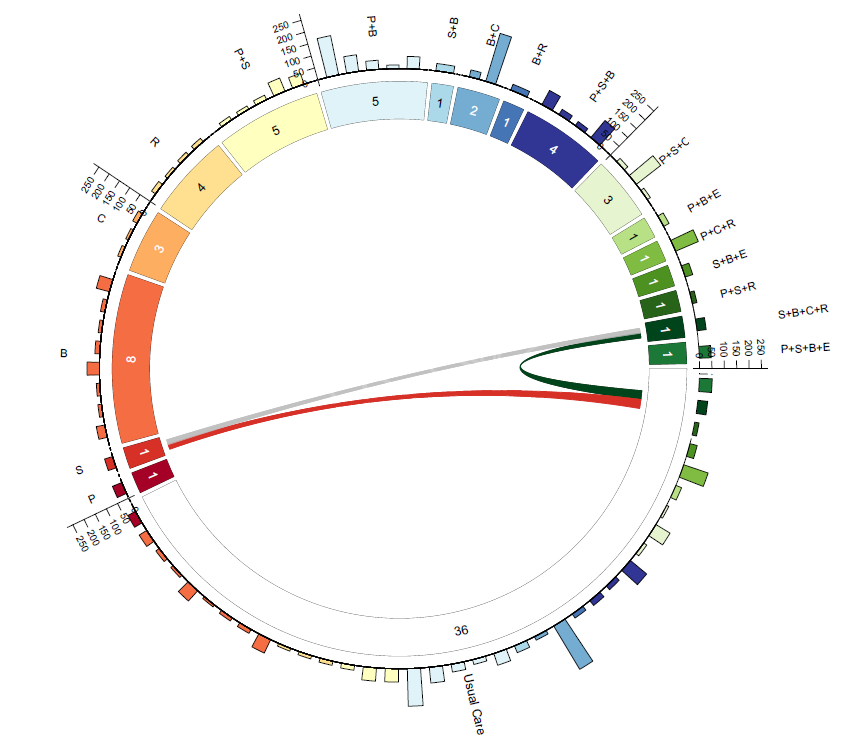


Figure S1: A single three-arm trial from the psychological preparations dataset. Links between interventions are coloured by the difference between arms. Links are coloured grey when the combination of components that differ between arms are not trialled in any single arm in the network. Multi-arm trials are denoted by multiple thinner connecting links. Numbers at the end of each link represent the number of trial arms for each intervention. Bars represent the sample size for each trial arm. In this network all studies included usual care. For trial arms including additional components, usual care has been excluded from the intervention label. E = emotion-focused techniques, R = relaxation, C = cognitive interventions, S = sensory information, P = procedural information, B = behavioural instruction.

**R code for Figure 1**

library(netmeta)

# Load data

data <- read.csv("length_of_stay.csv")

data2 <- pairwise(treat=treatment, n=N, mean=Mean, sd=SD,

studlab=ID, data=data, sm="MD")

net1 <- netmeta(TE, seTE, treat1, treat2, studlab, data=data2, sm="MD", comb.fixed=FALSE,

comb.random=TRUE)

# Specify order of treatments in plot

trts <- c("Usual Care", "P", "S", "B", "C", "R", "P+S", "P+B", "S+B", "B+C", "B+R", "P+S+B","P+S+C", "P+S+R", "P+B+E", "P+C+R", "S+B+E", "P+S+B+E", "S+B+C+R")

netgraph(net1, plastic=F, col="black", points=T,

col.points="blue", number.of.studies = F,

seq=trts, thickness="number.of.studies", cex.points=2,

offset=0.05, scale=0.7, cex=0.8)

**R code for Figure 2**

library(UpSetR)

# Load data

data <- read.csv("length_of_stay.csv")

upset(data, nsets = 7, nintersects = NA, number.angles = 0,

point.size = 2,

line.size = 0.7,

order.by = "freq",

matrix.color = "#1a5276",

main.bar.color = "#6c3483",

sets.bar.color = "#138d75",

set_size.show = TRUE,

mainbar.y.label = "No. of Trial Arms featuring combination",

sets.x.label = "No. of Trial Arms featuring component",

mainbar.y.max = 40,

text.scale = 1.5)

**R code for Figure 3**

library(corrplot)

# Read in study data

dat <- read.csv("length_of_stay.csv")

# Component labels

labdat <- data.frame(coding1 = c("P", "S", "B", "C", "R", "E"))

labs <- c("Procedural Information", "Sensory Information", "Behavioural Instruction",

"Cognitive Intervention", "Relaxation",

"Emotion-focused techniques")

# Generate object with data for plot - Which trial arms feature each component

datas <- vector("list",2)

for (i in 1:length(labdat$coding1)) {

x <- as.character(labdat$coding1[i])

lab <- as.character(labdat$coding1[i])

datas[[i]] <- rownames(subset(dat, get(x) == 1))

names(datas)[i] <- lab

rm(x,lab,i)

}

# Reduce data down to component columns and add component labels

comp <- labdat$coding1

x <- dat[,comp]

names(x) <- labs

# compute phi-coefficients:

y <- cor(x)

# create correlation plot:

corrplot(y, method = "color", type = "upper", diag = FALSE,

tl.cex = 1, tl.col = "black",

number.cex = 1, number.font = 2, number.digits = 2,

col=COL2("PRGn"), addCoef.col="black")

**R code for Figure 4**

library(circlize)

library(ComplexHeatmap)

# Read in study data

dat <- read.csv("length_of_stay.csv")

# Component labels

labdat <- data.frame(coding1 = c("P", "S", "B", "C", "R", "E"))

labs <- c("Procedural Information", "Sensory Information", "Behavioural Instruction",

"Cognitive Intervention", "Relaxation",

"Emotion-focused techniques")

# Generate object with data for plot - Which trial arms feature each component

datas <- vector("list",2)

for (i in 1:length(labdat$coding1)) {

x <- as.character(labdat$coding1[i])

lab <- as.character(labdat$coding1[i])

datas[[i]] <- rownames(subset(dat, get(x) == 1))

names(datas)[i] <- lab

rm(x,lab,i)

}

# Calculate number of components with each arm

comp <- labdat$coding1

x <- dat[,comp]

x$ncomps <- apply(x,1,sum)

# Number of components

ncomps <- length(comp)

# Set up empty matrix for matrix with number of pairs of component combinations

a <- matrix(nrow = ncomps, ncol = ncomps,

dimnames = list(labs,labs))

# Fill in the matrix:

for (i in 1: ncomps){

r <- x[,comp[i]]

for (j in 1:ncomps){

c <- x[,comp[j]]

a[i,j] <- with(x, sum(r==1 & c==1))

rm(j,c)

}

rm(i,r)

}

# Choose colour scale for heatmap

col_fun = colorRamp2(c(0, 30), c("white", "red"))

# Create lower triangle of a heatmap

Heatmap(a,

name = "N arms with\ncombination\n",

col = col_fun,

cluster_rows = FALSE,

cluster_columns = FALSE,

row_names_side = "left",

row_names_max_width = unit(10, "cm"),

row_names_gp = gpar(fontsize = 15),

column_names_side = "bottom",

column_names_max_height = unit(10, "cm"),

column_names_gp = gpar(fontsize = 15),

column_title_gp = gpar(fontsize = 16, fontface = "bold"),

rect_gp = gpar(type = "none"),

cell_fun = function(j, i, x, y, w, h, fill){

if( i>=j ){

grid.rect(x, y, w, h,

gp = gpar(fill = fill, col = fill))

grid.text(sprintf("%.0f", a[i, j]),

x, y,

gp = gpar(fontsize = 15))

}

}

)

**R code for Figure 5**

library(circlize)

mat = matrix(c(1,1,1,1,2,1,1,1,1,1,1,1,1),13,1)

rownames(mat)= c("Or+Mob","Or+Se+Cog", "Or+Nu+Mob", "Cog+Mob+Bo", "Or+Se+Nu+Mob+Sl", "Cog+Nu+Mob+Me+Mo", "Se+Nu+Sl+Ox+PC+Me+Bo", "Nu+Inf+Mob+Sl+Ox+PC+Bo", "Or+Se+Cog+Nu+Inf+Mob+Sl+PC", "Or+Se+Nu+Mob+Sl+Ox+PC+Me+Bo", "Or+Nu+Inf+Mob+Sl+Ox+PC+Me+Bo", "Or+Se+Cog+Nu+Inf+Mob+Ox+PC+Me+Bo", "Or+Se+Nu+Inf+Mob+Sl+PC+Me+Mo+Bo")

colnames(mat)= "Usual Care"

circos.clear()

circos.par("start.degree" = -20,gap.after = 5 ,track.margin= c(0.009,0.009),track.height=0.3,cell.padding = c(0.02,1.00, 0.02, 1.00), clock.wise =TRUE)

grid.col = c("Usual Care"="grey", "Or+Mob"="#a50026", "Or+Se+Cog"="#d73027", "Or+Nu+Mob"="#f46d43", "Cog+Mob+Bo"="red", "Or+Se+Nu+Mob+Sl"="#fee090", "Cog+Nu+Mob+Me+Mo"="#ffffbf", "Se+Nu+Sl+Ox+PC+Me+Bo"="#e0f3f8", "Nu+Inf+Mob+Sl+Ox+PC+Bo"="#abd9e9", "Or+Se+Cog+Nu+Inf+Mob+Sl+PC"="#74add1", "Or+Se+Nu+Mob+Sl+Ox+PC+Me+Bo"="#4575b4", "Or+Nu+Inf+Mob+Sl+Ox+PC+Me+Bo"="#313695", "Or+Se+Cog+Nu+Inf+Mob+Ox+PC+Me+Bo"="#e6f5d0", "Or+Se+Nu+Inf+Mob+Sl+PC+Me+Mo+Bo"="#b8e186")

chordDiagram(mat,grid.col = grid.col ,transparency = 0,

order = c("Usual Care", "Or+Mob", "Or+Se+Cog", "Or+Nu+Mob", "Cog+Mob+Bo", "Or+Se+Nu+Mob+Sl", "Cog+Nu+Mob+Me+Mo", "Se+Nu+Sl+Ox+PC+Me+Bo", "Nu+Inf+Mob+Sl+Ox+PC+Bo", "Or+Se+Cog+Nu+Inf+Mob+Sl+PC", "Or+Se+Nu+Mob+Sl+Ox+PC+Me+Bo", "Or+Nu+Inf+Mob+Sl+Ox+PC+Me+Bo", "Or+Se+Cog+Nu+Inf+Mob+Ox+PC+Me+Bo", "Or+Se+Nu+Inf+Mob+Sl+PC+Me+Mo+Bo"),

annotationTrack ="grid", annotationTrackHeight=convert_height(c(2,2),"mm"),preAllocateTracks = 1)

circos.trackPlotRegion(track.index=1, ylim = c(0,500), track.height=0.5, panel.fun = function(x, y) {

sector.index = get.cell.meta.data("sector.index")

})

circos.track(track.index = 1, sector= "Usual Care", panel.fun = function(x, y) {

value1=matrix(c(133,64,129,29,370,97,53,91,25,30,184,179,143,343,19,32,25,12,33,73,9,22,8,5,39,27,19,21),ncol=2)

circos.barplot(value1,1:14-0.5, col=c("grey","white","#b8e186","#e6f5d0","#313695","#4575b4","#74add1","#abd9e9","#e0f3f8","#ffffbf","#fee090","#fee090","red","#f46d43","#d73027","#a50026"))

circos.text(CELL_META$xcenter, CELL_META$ylim[1], CELL_META$sector.index,facing = "clockwise", niceFacing = TRUE, adj = c(-0.7,0.9),cex=1.1)

circos.axis(h = 0, major.at = 0,minor.ticks=1, labels.cex = 0.1)

},

bg.border = 0)

highlight.sector("Usual Care", track.index = 2,col="grey", text = "14", cex =1, text.col = "black", niceFacing = TRUE)

circos.track(track.index = 1, sector="Or+Mob", panel.fun = function(x, y) {

value2 = matrix(c(305,15), ncol=2)

circos.barplot(value2,1-0.5, col=c("#a50026","white"))

circos.text(CELL_META$xcenter, CELL_META$ylim[2], CELL_META$sector.index,facing = "clockwise", niceFacing = TRUE, adj = c(1,0.9),cex=0.9)

circos.axis(h = 0, major.at = 0, minor.ticks = 1,labels.cex =0.1)

},

bg.border = 0)

highlight.sector("Or+Mob", track.index = 2,col="#a50026", text = "1", cex = 1, text.col = "white", niceFacing = TRUE)

circos.track(track.index = 1, sector="Or+Se+Cog", panel.fun = function(x, y) {

value3 =matrix(c(144,8), ncol=2)

circos.barplot(value3, 1 - 0.5, col=c("#d73027","white"))

circos.text(CELL_META$xcenter, CELL_META$ylim[2], CELL_META$sector.index,facing = "clockwise", niceFacing = TRUE, adj = c(1.1,0.9),cex=0.9)

circos.axis(h = 0, major.at = 0, minor.ticks = 1,labels.cex =0.1)

},

bg.border = 0)

highlight.sector("Or+Se+Cog", track.index = 2,col="#d73027", text = "1", cex = 1, text.col = "white",niceFacing = TRUE)

circos.track(track.index = 1, sector="Or+Nu+Mob", panel.fun = function(x, y) {

value4 = matrix(c(196,13), ncol=2)

circos.barplot(value4, 1- 0.5 ,col=c("#f46d43","white"))

circos.text(CELL_META$xcenter, CELL_META$ylim[2], CELL_META$sector.index,facing = "clockwise", niceFacing = TRUE, adj = c(1.1,0.9),cex=0.9)

circos.axis(h = 0, major.at = 0, minor.ticks = 1,labels.cex =0.1)

},

bg.border = 0)

highlight.sector("Or+Nu+Mob", track.index = 2,col="#f46d43", text = "1", cex = 1, text.col = "white",niceFacing = TRUE)

circos.track(track.index = 1, sector="Cog+Mob+Bo", panel.fun = function(x, y) {

value5 = matrix(c(186,27), ncol=2)

circos.barplot(value5,1-0.5, col=c("red","white"))

circos.text(CELL_META$xcenter, CELL_META$ylim[2], CELL_META$sector.index,facing = "clockwise", niceFacing = TRUE, adj = c(1,0.9),cex=0.9)

circos.axis(h = 0, major.at = 0, minor.ticks = 1,labels.cex =0.1)

},

bg.border = 0)

highlight.sector("Cog+Mob+Bo", track.index = 2,col="red", text = "1", cex = 1, text.col = "white",niceFacing = TRUE)

circos.track(track.index = 1, sector="Or+Se+Nu+Mob+Sl", panel.fun = function(x, y) {

value6 = matrix(c(30,20,0,4),ncol=2)

circos.barplot(value6, 1:2-0.5, col=c("#fee090","white"))

circos.text(CELL_META$xcenter, CELL_META$ylim[2], CELL_META$sector.index,facing = "clockwise", niceFacing = TRUE, adj = c(1.1,0.9),cex=0.9)

circos.axis(h = 0, major.at = 0, minor.ticks = 1,labels.cex =0.1)

},

bg.border = 0)

highlight.sector("Or+Se+Nu+Mob+Sl", track.index = 2,col="#fee090", text = "2", cex = 1, text.col = "black",niceFacing = TRUE)

circos.track(track.index = 1, sector="Cog+Nu+Mob+Me+Mo", panel.fun = function(x, y) {

value7 = matrix(c(85,9), ncol=2)

circos.barplot(value7, 1-0.5, col=c("#ffffbf","white"))

circos.text(CELL_META$xcenter, 1, CELL_META$sector.index,facing = "clockwise", niceFacing = TRUE, adj = c(-0.3,0.9),cex=0.9)

circos.axis(h = 0, major.at = 0, minor.ticks = 1,labels.cex =0.1)

},

bg.border = 0)

highlight.sector("Cog+Nu+Mob+Me+Mo", track.index = 2,col="#ffffbf", text = "1", cex = 1, text.col = "black",niceFacing = TRUE)

circos.track(track.index = 1, sector="Se+Nu+Sl+Ox+PC+Me+Bo", panel.fun = function(x, y) {

value8 = matrix(c(50,2), ncol=2)

circos.barplot(value8, 1-0.5, col=c("#e0f3f8","white"))

circos.text(CELL_META$xcenter, 1, CELL_META$sector.index,facing = "clockwise", niceFacing = TRUE, adj = c(-0.2,0.9),cex=0.9)

circos.axis(h = 0, major.at =0, minor.ticks = 1,labels.cex =0.1)

},

bg.border = 0)

highlight.sector("Se+Nu+Sl+Ox+PC+Me+Bo", track.index = 2,col="#e0f3f8", text = "1", cex = 1, text.col = "black",niceFacing = TRUE)

circos.track(track.index = 1, sector="Nu+Inf+Mob+Sl+Ox+PC+Bo", panel.fun = function(x, y) {

value9 = matrix(c(102,56), ncol=2)

circos.barplot(value9, 1-0.5, col=c("#abd9e9","white"))

circos.text(CELL_META$xcenter, 1, CELL_META$sector.index,facing = "clockwise", niceFacing = TRUE, adj = c(-0.4,0.9),cex=0.9)

circos.axis(h = 0, major.at =0, minor.ticks = 1,labels.cex =0.1)

},

bg.border = 0)

highlight.sector("Nu+Inf+Mob+Sl+Ox+PC+Bo", track.index = 2,col="#abd9e9", text = "1", cex = 1, text.col = "black",niceFacing = TRUE)

circos.track(track.index = 1, sector="Or+Se+Cog+Nu+Inf+Mob+Sl+PC", panel.fun = function(x, y) {

value10 =matrix(c(343,24), ncol=2)

circos.barplot(value10, 1-0.5, col=c("#74add1","white"))

circos.text(0, 1, CELL_META$sector.index,facing = "clockwise", niceFacing = TRUE, adj = c(-0.6,5),cex=0.9)

circos.axis(h = 0, major.at = 0, minor.ticks = 1,labels.cex =0.1)

},

bg.border = 0)

highlight.sector("Or+Se+Cog+Nu+Inf+Mob+Sl+PC", track.index = 2,col="#74add1", text = "1", cex = 1, text.col = "white",niceFacing = TRUE)

circos.track(track.index = 1, sector="Or+Se+Nu+Mob+Sl+Ox+PC+Me+Bo", panel.fun = function(x, y) {

value11 = matrix(c(21,3), ncol=2)

circos.barplot(value11, 1-0.5, col=c("#4575b4","white"))

circos.text(CELL_META$xcenter, CELL_META$ylim[2], CELL_META$sector.index,facing = "clockwise", niceFacing = TRUE, adj = c(0.6,0.5),cex=0.9)

circos.axis(h = 0, major.at =0, minor.ticks = 1,labels.cex =0.1)

},

bg.border = 0)

highlight.sector("Or+Se+Nu+Mob+Sl+Ox+PC+Me+Bo", track.index = 2,col="#4575b4", text = "1", cex = 1, text.col = "white",niceFacing = TRUE)

circos.track(track.index = 1, sector="Or+Nu+Inf+Mob+Sl+Ox+PC+Me+Bo", panel.fun = function(x, y) {

value12 = matrix(c(152,4), ncol=2)

circos.barplot(value12, 1-0.5, col= c("#313695","white"))

circos.text(CELL_META$xcenter, CELL_META$ylim[2], CELL_META$sector.index,facing = "clockwise", niceFacing = TRUE, adj = c(0.4,0.9),cex=0.9)

circos.axis(h = 0, major.at =0, minor.ticks = 1,labels.cex =0.1)

},

bg.border = 0)

highlight.sector("Or+Nu+Inf+Mob+Sl+Ox+PC+Me+Bo", track.index = 2,col="#313695", text = "1", cex = 1, text.col = "white",niceFacing = TRUE)

circos.track(track.index = 1, sector="Or+Se+Cog+Nu+Inf+Mob+Ox+PC+Me+Bo", panel.fun = function(x, y) {

value13 = matrix(c(67,20), ncol=2)

circos.barplot(value13, 1-0.5, col= c("#e6f5d0","white"))

circos.text(CELL_META$xcenter, CELL_META$ylim[2], CELL_META$sector.index,facing = "clockwise", niceFacing = TRUE, adj = c(0.4,0.9),cex=0.9)

circos.axis(h = 0, major.at = 0, minor.ticks = 1,labels.cex =0.1)

},

bg.border = 0)

highlight.sector("Or+Se+Cog+Nu+Inf+Mob+Ox+PC+Me+Bo", track.index = 2,col="#e6f5d0", text = "1", cex = 1, text.col = "black",niceFacing = TRUE)

circos.track(track.index = 1, sector="Or+Se+Nu+Inf+Mob+Sl+PC+Me+Mo+Bo", panel.fun = function(x, y) {

value14 = matrix(c(127,12), ncol=2)

circos.barplot(value14, 1-0.5,col=c("#b8e186","white"))

circos.text(CELL_META$xcenter, CELL_META$ylim[2], CELL_META$sector.index,facing = "clockwise", niceFacing = TRUE, adj = c(0.4,0.9),cex=0.9)

circos.axis(h = 0, major.at = 0, minor.ticks = 1,labels.cex =0.1)

},

bg.border = 0)

highlight.sector("Or+Se+Nu+Inf+Mob+Sl+PC+Me+Mo+Bo", track.index = 2,col="#b8e186", text = "1", cex = 1, text.col = "black",niceFacing = TRUE)

par(cex = 0.65)

sectors=c("Usual Care","Or+Mob","Or+Se+Cog+Nu+Inf+Mob+Sl+PC","Or+Se+Nu+Mob+Sl")

for(a in sectors) {

circos.yaxis(side = "left", sector.index = a, track.index=1)

}

**R code for Figure 6**

# Load packages

library(readxl)

library(circlize)

library(tidyr)

library(dplyr)

# Read the data from the Excel file

data <- read.csv("length_of_stay.csv")

# Count the number of trials for each treatment

trial_counts <- table(data$treatment)

# Convert trial counts to a data frame

trial_counts_df <- data.frame(treatment = names(trial_counts),

trials = as.numeric(trial_counts))

# Define the order of treatment based on my dataset

trial_counts_df<- data.frame(treatment = c("Usual Care","P","S","B","C","R","P+S","P+B","S+B","B+C","B+R","P+S+B",

"P+S+C","P+B+E","P+C+R","S+B+E","P+S+R","S+B+C+R","P+S+B+E"),trials = c(36,1,1,8,3,4,5,5,1,2,1,4,3,1,1,1,1,1,1))

# Set up the circular plot

circos.par("start.degree"=0,gap.after = 1 ,track.margin= c(0.009,0.009),track.height=0.2,

cell.padding = c(0.02,1.00, 0.02, 1.00), clock.wise =TRUE)

# Define the sectors based on my order

sectors <- c("Usual Care","P","S","B","C","R","P+S","P+B","S+B","B+C","B+R","P+S+B",

"P+S+C","P+B+E","P+C+R","S+B+E","P+S+R","S+B+C+R","P+S+B+E")

start_pos = 0

end_pos = start_pos + trial_counts_df$trial[1]

# Initialize the matrix for the sector widths

xlim = matrix(nrow = length(unique(data$treatment)), ncol = 2)

# Loop over the treatments

for (i in 1:length(unique(data$treatment))) {

xlim[i, 1] = start_pos

xlim[i, 2] = end_pos

start_pos = end_pos

if (i < length(data$treatment)) {

end_pos = start_pos + trial_counts_df$trial[i + 1]

}

}

# Define the limits of the y-axis

ylim <- c(0, max(trial_counts))

circos.clear()

# Create the circular plot

circos.initialize(factors = sectors, xlim = xlim)

# Define the color of sectors based on Color Brewer

grid.col = c( "Usual Care"="white","P"="#a50026", "S" = "#d73027", "B" = "#f46d43",

"C" = "#fdae61", "R" = "#fee090", "P+S" = "#ffffbf",

"P+B" = "#e0f3f8", "S+B" = "#abd9e9", "B+C" = "#74add1",

"B+R" = "#4575b4", "P+S+B" = "#313695", "P+S+C" = "#e6f5d0",

"P+B+E" = "#b8e186", "P+C+R" = "#7fbc41", "S+B+E" = "#4d9221",

"P+S+R" = "#276419", "P+S+B+E" = "#00441b", "S+B+C+R" = "#1b7837")

# Get the unique treatments

unique_treatments <- sectors

# Create an empty list to store the number of patients for each unique treatment

num_patients <- list()

# Loop through the unique treatments and calculate the number of patients for each treatment

for (i in unique_treatments) {

num_patients[[i]] <- data$N[data$treatment == i]

}

# View the number of patients for each unique treatment

num_patients

sector.values<-num_patients

# Plot barplot for each sector

a <- matrix(c(0, 36, 36, 37, 37, 38, 38, 46, 46, 49, 49, 53, 53, 58, 58, 63, 63, 64, 64, 66,

66, 67, 67, 71, 71, 74, 74, 75, 75, 76, 76, 77, 77, 78, 78, 79, 79, 80), ncol = 2, byrow = TRUE)

# calculate the length of each sector

sector_length <- diff(a, axis = 1)

# calculate the cumulative sum of the sector lengths

cum_sector_length <- c(0, cumsum(sector_length))

# calculate the start position of each bar for each sector

start_pos <- cum_sector_length[-length(cum_sector_length)]

for (i in 1:length(sectors)){

if (sectors[i] == "Usual Care") {

bar_colors <- c("#1b7837","#00441b","#276419","#4d9221","#7fbc41","#b8e186","#e6f5d0","#e6f5d0",

"#e6f5d0","#313695","#313695","#313695","#4575b4","#74add1","#74add1","#abd9e9","#e0f3f8",

"#e0f3f8","#e0f3f8","#e0f3f8","#e0f3f8","#ffffbf","#ffffbf", "#ffffbf","#fee090",

"#fee090","#fee090","#f46d43","#f46d43","#f46d43","#f46d43","#f46d43","#f46d43","#f46d43",

"#f46d43","#f46d43","#a50026")

} else {

bar_colors <- grid.col[sectors[i]]

}

circos.track(track.index = 1, sector = sectors[i], ylim =c(0,200),

panel.fun = function(x, y) {

sector.index = get.cell.meta.data("sector.index")

circos.barplot(sector.values[[sectors[i]]],pos = start_pos[i]:(start_pos[i] + sector_length[i] - 1),col=bar_colors)

circos.text(CELL_META$xcenter, CELL_META$ylim[1],CELL_META$sector.index,facing = "clockwise",

niceFacing = TRUE, adj = c(-1.3,1),cex=1.2)

circos.axis(h = 0, major.at = 0,minor.ticks=1, labels.cex = 0.1)

}, bg.border = 0)

}

circos.trackPlotRegion(track.index=2, sector= sectors, ylim = c(0,200),track.height=0.1,bg.col=grid.col,

panel.fun = function(x, y) {

sector.index = get.cell.meta.data("sector.index")

})

bar_plot_counts <- c(36,1,1,8,3,4,5,5,1,2,1,4,3,1,1,1,1,1,1)

# Initialize a variable to store the previous sector color

prev_color <- NA

for (i in 1:length(sectors)) {

# Set the color to the previous sector color

col <- ifelse(is.na(prev_color), "white", prev_color)

# Plot the sector and store the color

prev_color <- highlight.sector(sectors[i], track.index = 2, col = col,

text = bar_plot_counts[i], cex = 1.2,

text.col = "black", border.col = NA,

niceFacing = TRUE)$col

}

# Plot the links between interventions

circos.link("Usual Care", c(0,1), "P+S+B+E",c(79,80),col = c("#1b7837", transparency = 0),lwd=3)

circos.link("Usual Care", c(3,4), "S+B+E",c(76,77),col = c("#4d9221", transparency = 0),lwd=3)

circos.link("Usual Care", c(4,5), "P+C+R",c(75,76),col = c("#7fbc41", transparency = 0),lwd=3)

circos.link("Usual Care", c(5,6), "P+B+E",c(74,75),col = c("#b8e186", transparency = 0),lwd=3)

circos.link("Usual Care", c(6,7), "P+S+C",c(73,74),col = c("#e6f5d0", transparency = 0))

circos.link("Usual Care", c(9,11), "P+S+B",c(69,71),col = c("#313695", transparency = 0))

circos.link("Usual Care", c(12,13), "B+R",c(66,67),col = c("#4575b4", transparency = 0),lwd=3)

circos.link("Usual Care", c(13,15), "B+C",c(64,66),col = c("#74add1", transparency = 0))

circos.link("Usual Care", c(15,16), "S+B",c(63,64),col = c("#abd9e9", transparency = 0))

circos.link("Usual Care", c(16,21), "P+B",c(58,63),col = c("#e0f3f8", transparency = 0))

circos.link("Usual Care", c(21,23), "P+S",c(56,58),col = c("#ffffbf", transparency = 0))

circos.link("Usual Care", c(24,27), "R",c(49,52),col = c("#fee090", transparency = 0))

circos.link("Usual Care", c(27,35), "B",c(38,46),col = c("#f46d43", transparency = 0))

circos.link("Usual Care", c(35,36), "P",c(36,37),col = c("#a50026", transparency = 0),lwd=2)

circos.link("Usual Care", c(11,11.5), "P+S+B",c(68.5,69),col = c("#313695CC", alpha=0.5))

circos.link("Usual Care", c(11.5,12), "C",c(47,47.5),col = c("#fdae61CC", alpha=0.5))

circos.link("C", c(47.5,48), "P+S+B",c(68,68.5),col = "grey")

circos.link("Usual Care", c(1,1.5), "S+B+C+R",c(78.5,79),col = c("#00441b", alpha=0.5))

circos.link("Usual Care", c(1.5,2), "S",c(37,37.5),col = c("#d73027", alpha=0.5))

circos.link("S", c(37.5,38), "S+B+C+R",c(78,78.5),col = "grey")

circos.link("Usual Care", c(23,23.5), "P+S",c(55.5,56),col = c("#ffffbf", alpha=0.5))

circos.link("Usual Care", c(23.5,24), "C",c(48,48.5),col = c("#fdae61", alpha=0.5))

circos.link("C", c(48.5,49), "P+S",c(55,55.5),col = "grey")

circos.link("Usual Care", c(7,7.5), "P+S+C",c(72.5,73),col = c("#e6f5d0", alpha = 0.5))

circos.link("Usual Care", c(7.5,8), "P+S+B",c(67,67.5),col = c("#313695",alpha= 0.5))

circos.link("P+S+B", c(67.5,68), "P+S+C",c(72,72.5),col = "grey")

circos.link("Usual Care", c(2,2.3), "P+S+R",c(77.6,78),col = c("#276419", transparency = 0.5))

circos.link("Usual Care", c(2.3,2.6), "P+S",c(53,53.3),col = c("#ffffbf", transparency = 0.5))

circos.link("Usual Care", c(2.6,3), "R",c(52,52.3),col = c("#fee090", transparency = 0.5))

circos.link("P+S", c(53.3,53.6), "P+S+R",c(77,77.3),col = c("#fee090", transparency = 0.5))

circos.link("R", c(52.3,52.6), "P+S+R",c(77.3,77.6),col = c("#ffffbf", transparency = 0.5))

circos.link("R", c(52.6,53), "P+S",c(53.6,54),col = c("dark grey", transparency = 0.5))

circos.link("Usual Care", c(8,8.3), "P+S+C",c(71.6,72),col = c("#e6f5d0", transparency = 0))

circos.link("Usual Care", c(8.3,8.6), "P+S",c(54.3,54.6),col = c("#ffffbf", transparency = 0))

circos.link("Usual Care", c(8.6,9), "C",c(46,46.3),col = c("#fdae61", transparency = 0))

circos.link("P+S", c(54.6,55), "P+S+C",c(71,71.3),col = c("#fdae61", transparency = 0))

circos.link("C", c(46.3,46.6), "P+S+C",c(71.3,71.6),col = c("#ffffbf", transparency = 0))

circos.link("C", c(46.6,47), "P+S",c(54,54.3),col = c("grey", transparency = 0))

par(cex = 0.75)

sectors=c("Usual Care","P","R","P+B","P+S+C")

for(a in sectors) {

circos.yaxis(side = "left", sector.index = a, track.index=1)

}

**References**

1. Kossmeier M, Tran US, Voracek M. Charting the landscape of graphical displays for meta-analysis and systematic reviews: a comprehensive review, taxonomy, and feature analysis. BMC Medical Research Methodology. 2020;20(1):26.

2. Hartmann-Boyce J, Livingstone-Banks J, Ordóñez-Mena JM, Fanshawe TR, Lindson N, Freeman SC, et al. Behavioural interventions for smoking cessation: an overview and network meta-analysis. Cochrane Database of Systematic Reviews. 2021.

3. Mills EJ, Druyts E, Ghement I, Puhan MA. Pharmacotherapies for chronic obstructive pulmonary disease: a multiple treatment comparison meta-analysis. 2011;3:107-129

4. Rücker G, Petropoulou M, Schwarzer G. Network meta-analysis of multicomponent interventions. Biometrical Journal. 2020;62(3):808–21.

5. Lopez-Lopez JA, Davies SR, Caldwell DM, Churchill R, Peters TJ, Tallon D, et al. The process and delivery of CBT for depression in adults: a systematic review and network meta-analysis. Psychological Medicine. 2019;49(12):1937–47.

6. Petropoulou M, Efthimiou O, Rücker G, Schwarzer G, Furukawa TA, Pompoli A, et al. A review of methods for addressing components of interventions in meta-analysis. Plos one. 2021;16(2):e0246631.

7. Launder N, Lampit A. Computerised cognitive training in cognitively healthy older adults: A systematic review and component network meta-analysis. Available from: https://scholar.archive.org/work/mgmfnahnmfajffwdvs4ukvamiq/access/wayback/https://objects.storage.unimelb.edu.au/2016UOM009:figshare/27245975/BIOM30003_NathalieLaunder_831935_MainPaper.pdf

8. Rücker G, Schmitz S, Schwarzer G. Component network meta-analysis compared to a matching method in a disconnected network: A case study. Biometrical Journal. 2021;6:447-461

9. Shi Q, Tan L, Chen Z, Ge L, Zhang X, Yang F, et al. Comparative Efficacy of Pharmacological and Nonpharmacological Interventions for Acne Vulgaris: A Network Meta-Analysis. Frontiers in pharmacology. 2020;11:1809.

10. Caldwell DM, Welton NJ. Approaches for synthesising complex mental health interventions in meta-analysis. Evidence-Based Mental Health. 2016;19(1):16–21.

11. Weibel S, Rücker G, Eberhart LH, Pace NL, Hartl HM, Jordan OL, et al. Drugs for preventing postoperative nausea and vomiting in adults after general anaesthesia: a network meta-analysis. Cochrane Database of Systematic Reviews. 2020.

12. Danko KJ. Methods for Optimizing Evidence Syntheses of Complex Interventions: Case Study of a Systematic Review and Meta-Analysis of Diabetes Quality Improvement Trials [PhD Thesis]. Université d’Ottawa/University of Ottawa; 2018. Available from: https://ruor.uottawa.ca/handle/10393/38225

13. Burton JK, Siddiqi N, Teale EA, Barugh A, Sutton AJ. Non-pharmacological interventions for preventing delirium in hospitalised non-ICU patients. The Cochrane Database of Systematic Reviews. 2019.

14. Freeman SC, Scott NW, Powell R, Johnston M, Sutton AJ, Cooper NJ. Component network meta-analysis identifies the most effective components of psychological preparation for adults undergoing surgery under general anesthesia. Journal of Clinical Epidemiology. 2018;98:105–16.

15. Kabboul NN, Tomlinson G, Francis TA, Grace SL, Chaves G, Rac V, et al. Comparative Effectiveness of the Core Components of Cardiac Rehabilitation on Mortality and Morbidity: A Systematic Review and Network Meta-Analysis. Journal of Clinical Medicine. 2018;7(12):514.

16. Salanti G, Ades AE, Ioannidis JPA. Graphical methods and numerical summaries for presenting results from multiple-treatment meta-analysis: an overview and tutorial. Journal of Clinical Epidemiology. 2011;64(2):163–71.

17. Rücker G, Schwarzer G. Ranking treatments in frequentist network meta-analysis works without resampling methods. BMC Medical Research Methodology. 2015;15(1):58.

18. Mbuagbaw L, Rochwerg B, Jaeschke R, Heels-Andsell D, Alhazzani W, Thabane L, et al. Approaches to interpreting and choosing the best treatments in network meta-analyses. Systematic Reviews. 2017;6(1):79.

19. Sposito AC, Bonilha I, Luchiari B, Benchimol A, Hohl A, Moura F, et al. Cardiovascular safety of naltrexone and bupropion therapy: Systematic review and meta-analyses. Obesity Reviews. 2021;22(6):e13224.

20. Wang SY, Hung YL, Hsu CC, Hu CH, Huang RY, Sung CM, et al. Optimal Perioperative Nutrition Therapy for Patients Undergoing Pancreaticoduodenectomy: A Systematic Review with a Component Network Meta-Analysis. Nutrients. 2021;13(11):4049.

21. Fong KM, Au SY, Ng GWY. Steroid, ascorbic acid, and thiamine in adults with sepsis and septic shock: a systematic review and component network meta-analysis. Scientific reports. 2021;11(1):1–7.

22. Cintra RM, Nogueira AC, Bonilha I, Luchiari BM, Coelho-Filho OR, Coelho OR, et al. Glucose-lowering Drugs and Hospitalization for Heart Failure: A Systematic Review and Additive-effects Network Meta-analysis With More Than 500 000 Patient-years. The Journal of Clinical Endocrinology & Metabolism. 2021;106(10):3060–7.

23. Chen YF, Madan J, Welton N, Yahaya I, Aveyard P, Bauld L, et al. Effectiveness and cost-effectiveness of computer and other electronic aids for smoking cessation: a systematic review and network meta-analysis. Health Technology Assessment. 2012;16(38):1.

24. Fujii T, Salanti G, Belletti A, Bellomo R, Carr A, Furukawa TA, et al. Effect of adjunctive vitamin C, glucocorticoids, and vitamin B1 on longer-term mortality in adults with sepsis or septic shock: a systematic review and a component network meta-analysis. Intensive care medicine. 2021;1–9.

25. Pompoli A, Furukawa TA, Efthimiou O, Imai H, Tajika A, Salanti G. Dismantling cognitive-behaviour therapy for panic disorder: a systematic review and component network meta-analysis. Psychological Medicine. 2018;48(12):1945–53.

26. Melton H, Meader N, Dale H, Wright K, Jones-Diette J, Temple M, et al. Interventions for adults with a history of complex traumatic events: the INCiTE mixed-methods systematcic review. Health Technology Assessment. 2020;24(43):1–312.

27. Roberts D, Best LM, Freeman SC, Sutton AJ, Cooper NJ, Arunan S, et al. Treatment for bleeding oesophageal varices in people with decompensated liver cirrhosis: a network meta‐analysis. Cochrane Database of Systematic Reviews. 2021.

28. Miklowitz DJ, Efthimiou O, Furukawa TA, Scott J, McLaren R, Geddes JR, et al. Adjunctive Psychotherapy for Bipolar Disorder: A Systematic Review and Component Network Meta-analysis. JAMA Psychiatry. 2020;78(2).

29. Welton NJ, Caldwell DM, Adamopoulos E, Vedhara K. Mixed Treatment Comparison Meta-Analysis of Complex Interventions: Psychological Interventions in Coronary Heart Disease. American Journal of Epidemiology. 2009;169(9):1158–65.

30. Madan J, Chen YF, Aveyard P, Wang D, Yahaya I, Munafo M, et al. Synthesis of evidence on heterogeneous interventions with multiple outcomes recorded over multiple follow-up times reported inconsistently: a smoking cessation case-study. Journal of the Royal Statistical Society Series A. 2014;177(1):295–314.

31. Mills EJ, Thorlund K, Ioannidis JPA. Calculating additive treatment effects from multiple randomized trials provides useful estimates of combination therapies. Journal of Clinical Epidemiology. 2012;65(12):1282–8.

32. Riemsma R, Lhachimi SK, Armstrong N, van Asselt T, Allen A, Manning N, et al. Roflumilast for the management of severe chronic obstructive pulmonary disease: a single technology appraisal. Kleijnen Systematic Reviews Ltd. 2011; Available from: https://www.academia.edu/1011173/Roflumilast_for_the_management_of_severe_chronic_obstructive_pulmonary_disease

33. Smith EA, Cooper NJ, Sutton AJ, Abrams KR, Hubbard SJ. A review of the quantitative effectiveness evidence synthesis methods used in public health intervention guidelines. BMC Public Health. 2021;21(1):278.

34. Dautzenberg L, Beglinger S, Tsokani S, Zevgiti S, Raijmann RC, Rodondi N, et al. Interventions for preventing falls and fall-related fractures in community-dwelling older adults: A systematic review and network meta-analysis. Journal of the American Geriatrics Society. 2021;69(10):2973–84.

35. Dautzenberg L, Bretagne L, Koek HL, Tsokani S, Zevgiti S, Rodondi N, et al. Medication review interventions to reduce hospital readmissions in older people. Journal of the American Geriatrics Society. 2021;69(6):1646–58.

36. Bálint A, Tornyos D, El Alaoui El Abdallaoui O, Kupó P, Komócsi A. Network Meta-Analysis of Ticagrelor for Stroke Prevention in Patients at High Risk for Cardiovascular or Cerebrovascular Events. Stroke. 2021;52(9):2809–16.

37. Veroniki AA, Seitidis G, Nikolakopoulos S, Ballester M, Beltran J, Heijmans M, et al. Modeling Multicomponent Interventions in Network Meta-Analysis. In: Evangelou E, Veroniki AA, editors. Meta-Research: Methods and Protocols. New York, NY: Springer US; 2022 [cited 2022 Jan 26]. p. 245–61. (Methods in Molecular Biology). Available from: https://doi.org/10.1007/978-1-0716-1566-9_15
